# Adjuvant-induced macrophage activation compromises BA71ΔCD2-mediated protection against African swine fever virus

**DOI:** 10.1038/s41541-026-01461-5

**Published:** 2026-04-24

**Authors:** Aida Tort-Miró, Sergio Montaner-Tarbes, David Marín-Moraleda, Jordana Muñoz-Basagoiti, Yan Zeng, María Jesús Navas, Marta Muñoz, Paula Monleon, Judith González-Oliver, Beatriz Martín-Mur, Marc Caballé, Margalida Artigues, Anna Esteve-Codina, Virginia Aragon, Enric Vidal, Àlex Cobos, Francesc Accensi, Sonia Pina-Pedrero, Elena Garcia-Fruitós, Fernando Rodríguez, Jordi Argilaguet

**Affiliations:** 1 Unitat Mixta d’Investigació IRTA-UAB en Sanitat Animal, Centre de Recerca en Sanitat Animal (CReSA), Campus de la Universitat Autònoma de Barcelona (UAB), Bellaterra, Catalonia Spain; 2 Institut de Recerca i Tecnologia Agroalimentàries (IRTA), Programa de Sanitat Animal, Centre de Recerca en Sanitat Animal (CReSA), Campus de la Universitat Autònoma de Barcelona (UAB), Bellaterra, Spain; 3 WOAH Collaborating Centre for the Research and Control of Emerging and Re-Emerging Swine Diseases in Europe (IRTA-CReSA), Bellaterra, Spain; 4 College of Veterinary Medicine, Jilin Agricultural University, Changchun, China; 5 Centro Nacional de Análisis Genómico (CNAG), Barcelona, Spain; 6 Universitat de Barcelona (UB), Barcelona, Spain; 7 IRTA, Valorization Office, Torre Marimon, Caldes de Montbui, Catalonia Spain; 8 Departament de Química Analítica i Aplicada, Institut Químic de Sarrià (IQS), Universitat Ramon Llull (URL), Barcelona, Spain; 9 Departament de Sanitat i d’Anatomia Animals, Facultat de Veterinària, Campus de la Universitat Autònoma de Barcelona (UAB), Bellaterra, Catalonia Spain; 10 IRTA, Ruminant Production, Torre Marimon, Caldes de Montbui, Catalonia Spain

**Keywords:** Immunology, Microbiology

## Abstract

While effective subunit vaccines against African swine fever (ASF) are still under development, live attenuated vaccines (LAVs) remain the only approach capable of inducing robust protective immunity, though biosafety concerns limit their field use. Thus, further research is required to improve LAV safety while maintaining its immunogenicity. Because both the ASF virus (ASFV) and derived LAVs suppress macrophage innate responses, we hypothesized that adjuvants could restore functionality of LAV-infected cells, allowing dose reduction and consequently minimizing the risk of adverse events. To test this, we intranasally vaccinated pigs with a suboptimal dose of the LAV BA71ΔCD2, either alone or combined with two immunostimulatory adjuvants derived from *Rothia nasimurium*. Both immunostimulants enhanced the in vitro responsiveness of BA71ΔCD2-infected macrophages, which acquired antigen-presenting features. However, in vivo, both adjuvants lowered humoral and cellular responses induced by BA71ΔCD2, consequently decreasing protection against lethal challenge. Further in vitro analyses demonstrated that adjuvant-activated macrophages acquired an antiviral state, limiting LAV replication. These results suggest the hypothesis that reduced vaccine efficacy might result from insufficient antigen production. Overall, the study indicates that combining adjuvants with ASFV-based LAVs would require a fine-tune macrophage activation to enhance functionality without excessively inhibiting viral replication.

## Introduction

The development of a safe and effective African swine fever (ASF) vaccine remains a major global challenge. The disease is one of the biggest threats affecting the pig industry worldwide, with a complex epidemiological pattern in Africa, and currently spreading in Asia and Europe through the infection of domestic pigs and wild boars (https://efsa.onlinelibrary.wiley.com/doi/10.2903/j.efsa.2023.8016). While the development of subunit vaccines is progressing^1,2^, it continues facing important limitations due to the lack of knowledge on protective antigens and immune responses^3,4^. In contrast, several experimental live attenuated vaccines (LAVs) confer robust immunity and protection against lethal challenge^5^. However, biosafety concerns hold back their implementation in the field^6^. Indeed, two LAVs were approved in Vietnam in 2022^7^, but their widespread usage is not yet a reality due to the potential of reversion to virulence and the emergence of new recombinant strains^8–11^. Thus, while waiting for the generation of safe and effective subunit vaccines, the development of novel LAV-based strategies with enhanced biosafety profiles might boost their application in ASF endemic areas.

The main target of the ASF virus (ASFV) is monocytes/macrophages. With more than 150-200 encoded proteins^12^, the virus has a high capability to hijack the infected cells, modulating their functional responses and eventually causing fatal cytopathic effects^13^. Indeed, throughout its replication cycle, ASFV modulates cytokine production, antigen presentation pathways and apoptosis- or pyroptosis-programmed cell death in infected cells^14,15^. Consequently, macrophages are unable to act as antigen-presenting cells (APC), not providing the required signals to other immune cells for a proper induction of a protective adaptive immune response. To note, many LAVs or naturally attenuated ASFV strains inducing protective immunity trigger higher innate immune responses in infected macrophages than the parental virulent strains^14,16,17^. This indicates that protective immunity induced by live ASFV-based immunisation is associated with the preservation of some degree of functionality in infected macrophages. However, the balance between virus attenuation and immunogenicity is subtle, and the generation of LAVs achieving a trade-off between biosafety and efficacy is complex. Notably, attempts to obtain safer LAVs through multiple gene deletion quite often result in reduced immunogenicity and loss of protective immunity^18–23^.

The use of adjuvants has usually been restricted to subunit or inactivated vaccines, which commonly have a weak intrinsic capability to stimulate immune responses^24^. In contrast, LAVs have an inherent ability to activate the innate immunity upon their recognition by pattern recognition receptors (PRRs), thus promoting the induction of vaccine-specific adaptive immune responses^25^. However, a few experimental studies have evaluated the co-administration of LAVs with adjuvants to test their ability to improve vaccine efficacy. In some cases, the combination of adjuvants with live vaccines has shown positive results, such as reduced vaccine side effects following vaccination with live *Leishmania major* combined with CpG DNA^26,27^, and sterilising immunity against pathogenic simian human immunodeficiency virus (SHIV) in macaques vaccinated with a live attenuated SHIV expressing the adjuvant Ag85B^28^. However, further research is required to evaluate the potential use of adjuvants for LAVs, since the effects will be highly adjuvant- and context-dependent. For instance, while the adjuvant α-C-galactosylceramide improved immune responses and protection in mice vaccinated with live attenuated influenza virus^29^, another study in pigs showed that the closely related adjuvant α-galactosylceramide interfered with heterotypic cross-protective immune responses^30^.

Here, we hypothesised that adjuvant-mediated activation of macrophages during ASF LAV immunisation would enhance vaccine efficacy, allowing for the reduction of the vaccine dose required to induce a protective immunity, and consequently improving its biosafety. To test this, we used the ASF BA71ΔCD2 LAV prototype as a model. This vaccine confers partial protection against a lethal challenge when pigs are immunised intranasally with a suboptimal dose^31^. As adjuvants, we used two different formulations derived from *Rothia nasimurium*, a bacterium with immunostimulatory properties that robustly activates porcine alveolar macrophages (PAMs)^32^. The results demonstrated that despite the enhanced responsiveness of macrophages induced by these adjuvants, including improved antigen-presenting cell features in vitro, their administration with the BA71ΔCD2 did not improve vaccine immunogenicity and efficacy in vivo. Indeed, the use of the adjuvants lowered vaccine-induced humoral and cellular responses, and the degree of protection against a challenge with a virulent ASFV strain. Importantly, further in vitro analyses showed a lower replication capacity of BA71ΔCD2 in adjuvant-activated macrophages, supporting a plausible model in which broad macrophage activation induces antiviral pathways that limit LAV replication and reduce antigen availability. These results represent a step forward in the understanding of early processes underlying LAV-induced immune protection, which might eventually help to rationally improve their biosafety profile.

## Results

### *Rothia nasimurium*-derived immunostimulants overcome the lack of responsiveness of BA71ΔCD2-infected macrophages

**Fig. 1:**
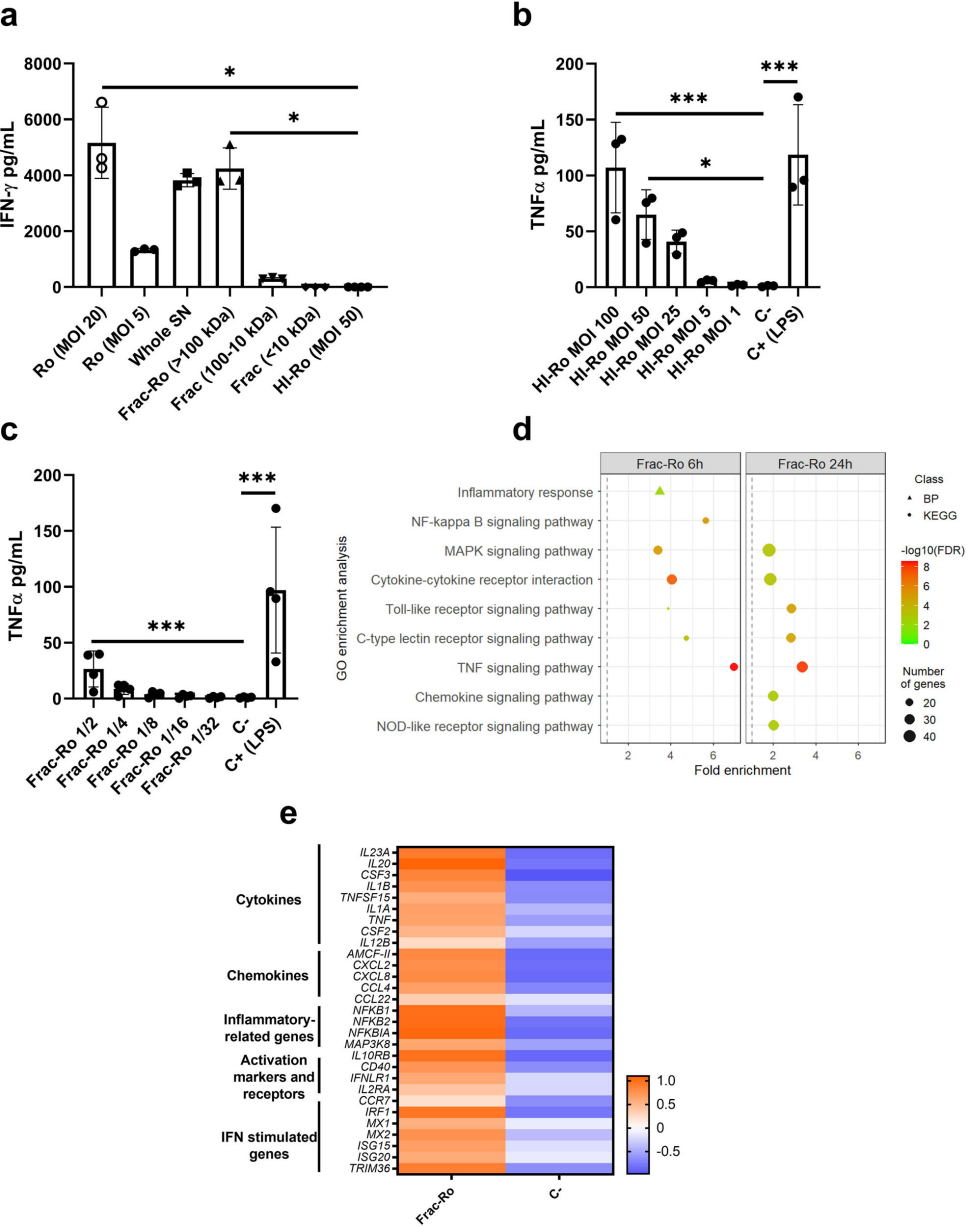
The cell-free supernatant from *Rothia nasimurium* cultures retains immunostimulatory properties. **a** Porcine alveolar macrophages (PAMs) were stimulated for 24 hours (h) with alive *Rothia nasimurium* (Ro), the whole supernatant (Whole SN), three different fractions of the supernatant separated by molecular sizes (Frac-Ro>100 kDa, Frac 100-10 kDa, Frac<10 kDa), or heat-inactivated *Rothia* (HI-Ro) at the indicated multiplicity of infection (MOI). **b**, **c** PAMs were stimulated with different doses of HI-Ro (MOI 100, 50, 25, 5, and 1), Frac-Ro (fraction containing components with molecular weight > 100 kDa; dilution 1/2, 1/4, 1/8, 1/16, 1/32), LPS (1 ug/mL), or left untreated for 24 hours. Levels of IFN-γ (**a**) or TNF (**b**, **c**) in cell supernatants were measured by ELISA. **d**, **e** PAMs were stimulated for 6 or 24 h with Frac-Ro, and their transcriptomic signature was obtained by RNA-seq. **d** List of representative Gene Ontology (GO) terms enriched among differentially expressed (DE) genes. **e** Heatmap depicting the z-score normalized log2CPM values from representative genes DE at 24 h post-stimulation in the indicated categories. Non-stimulated cells were used as a reference. **a**, **c** Significant differences were determined using a one-way ANOVA (*p-value ≤ 0.05, **≤ 0.01, ***≤ 0.001).

We have recently characterized the immunostimulatory properties of heat-inactivated *Rothia nasimurium* (HI-Ro), demonstrating its capability to activate antigen-presenting cells, thus showing its potential as a vaccine adjuvant^32^. Here, aiming to obtain a more purified product, we investigated whether the immunostimulatory components were present in cell-free supernatants from *R. nasimurium* culture. Indeed, stimulation of PAMs with supernatants induced IFN-γ, IL-12, and IL-1β secretion (Fig. 1a, Supplementary Fig. 1). Notably, the stimulatory capacity was only observed in a fraction of the supernatant containing components with a molecular weight >100 kDa (Frac-Ro), and not in fractions containing smaller components. To determine the optimal concentrations of HI-Ro and Frac-Ro for subsequent in vitro characterization, we performed a dose-response experiment in which PAMs were stimulated with decreasing concentrations of each *Rothia*-derived component. HI-Ro induced robust TNF production at a multiplicity of infection (MOI) of 50, without detectable toxicity (Fig. 1b, Supplementary Fig. 1h), and this concentration was selected for downstream assays. In contrast, Frac-Ro induced lower levels of TNF overall and only at the highest concentration obtainable from the extraction procedure (Fig. 1c, Supplementary Fig. 1i).

Interestingly, in contrast to Frac-Ro, HI-Ro did not stimulate IFN-γ production in macrophages (Fig. 1a), while both products induced robust TNF production (Fig. 1b, c). The stimulatory capacity of Frac-Ro was further demonstrated by analysing the transcriptome of treated macrophages by RNA-sequencing. Gene Ontology (GO) analysis of differentially expressed genes showed an enrichment in terms related to innate immunity, such as NF-κβ, MAPK, TLR and C-type lectin signalling pathways (Fig. 1d). Despite the differences mentioned above regarding IFN-γ production, this transcriptomic signature was very similar to HI-Ro- and lipopolysaccharide (LPS)-treated macrophages^32^, upregulating genes encoding for cytokines and chemokines, factors involved in inflammatory pathways, activation markers and receptors, and interferon stimulated genes (Fig. 1e). Indeed, 88% of the differentially expressed genes in Fra-Ro-treated macrophages were also identified in HI-Ro-stimulated cells, including genes enriched in several key innate immune pathways. However, HI-Ro induced the expression of a significantly larger number of genes than Frac-Ro, consistent with its more complex molecular composition (Supplementary Fig. 2).

Finally, we performed high-resolution mass spectrometry to characterize the molecular components present in Frac-Ro, aiming to identify the presence of potential immunostimulants. As shown in Supplementary Fig. 3a, direct infusion MS analysis revealed two predominant ion clusters in the regions of approximately 500–600 Da and 800–900 Da. Closer analysis of the 800–900 Da region (Supplementary Fig. 3b) demonstrated a series of ions separated by 14.01 Da, consistent with incremental differences in –CH₂ units within aliphatic chains. The observed ions (e.g., m/z 915.6042, 901.5911, 887.5731, 873.5629) were assigned to sodium adducts with mass errors below 10 ppm, supporting the presence of a homologous series of glycolipids with closely related structures but variable fatty acyl chain lengths. In contrast, ions detected in the 500–600 Da region (Supplementary Fig. 3c) showed mass errors greater than 10 ppm relative to the proposed sodium adducts and could not be confidently assigned to the same glycolipid family.

**Fig. 2:**
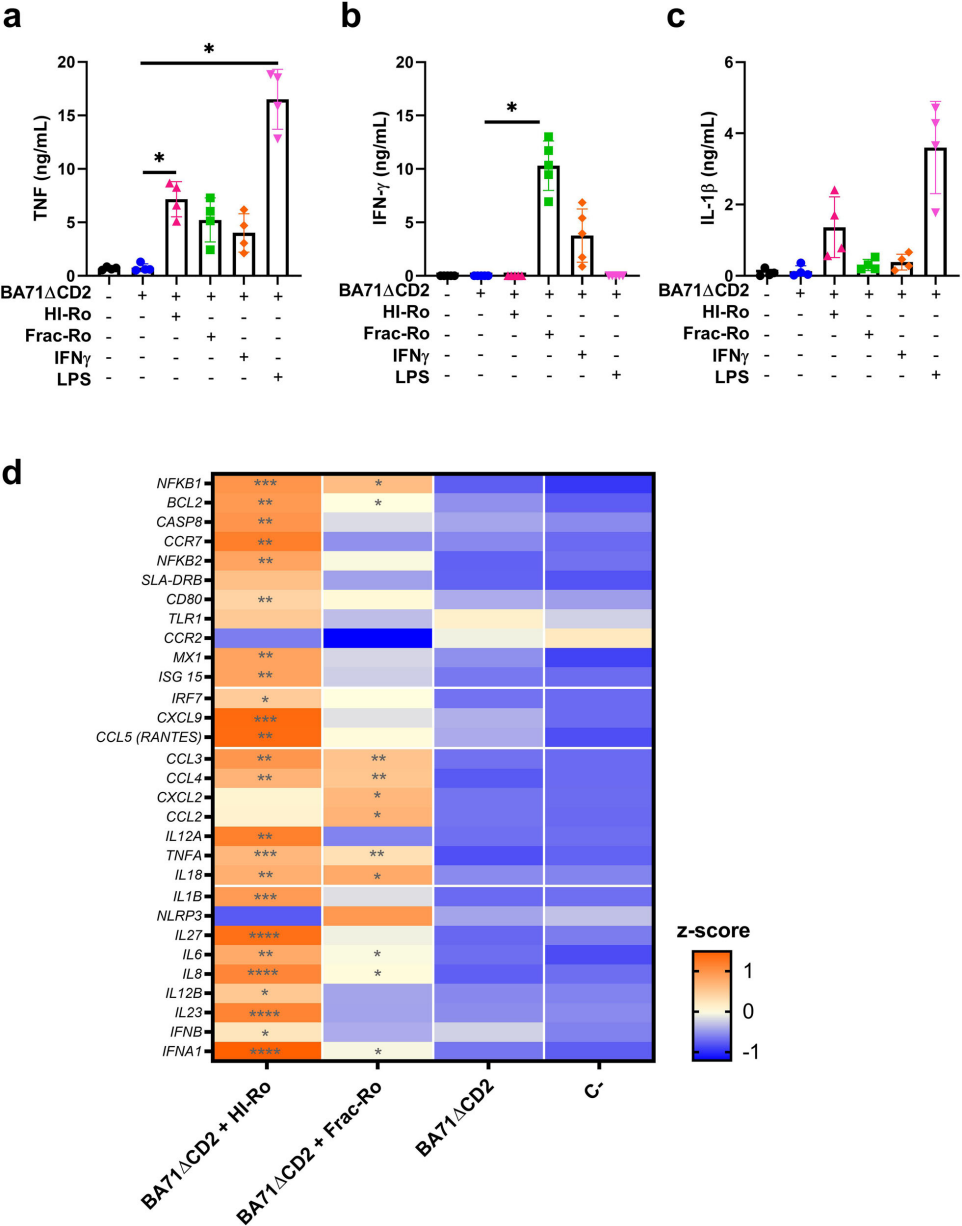
HI-Ro and Frac-Ro boost the functionality of BA71ΔCD2-infected macrophages. **a**–**d** Porcine alveolar macrophages (PAMs) were infected with the vaccine prototype BA71ΔCD2 for 2 h at MOI 0.1, and then treated with HI-Ro (MOI 50), Frac-Ro, IFN-γ (0.1 μg/mL), LPS (10 μg/mL), or left untreated. At 72 h post-infection, levels of TNF (**a**), IFN-γ (**b**), and IL-1β (**c**) were quantified by ELISA in supernatants. Non-infected cells were used as a control. **d** Expression levels of 30 genes representative of innate immune responses were quantified at 24 h post-infection by microfluidic quantitative PCR assay. The heatmap shows z-score normalized gene expression. Significant differences were determined using one-way ANOVA (**a**–**c**) or t-test comparing infected and non-infected PAMs (**d**) (*p-value ≤ 0.05, **≤ 0.01, ***≤ 0.001, and ****≤0.0001).

**Fig. 3:**
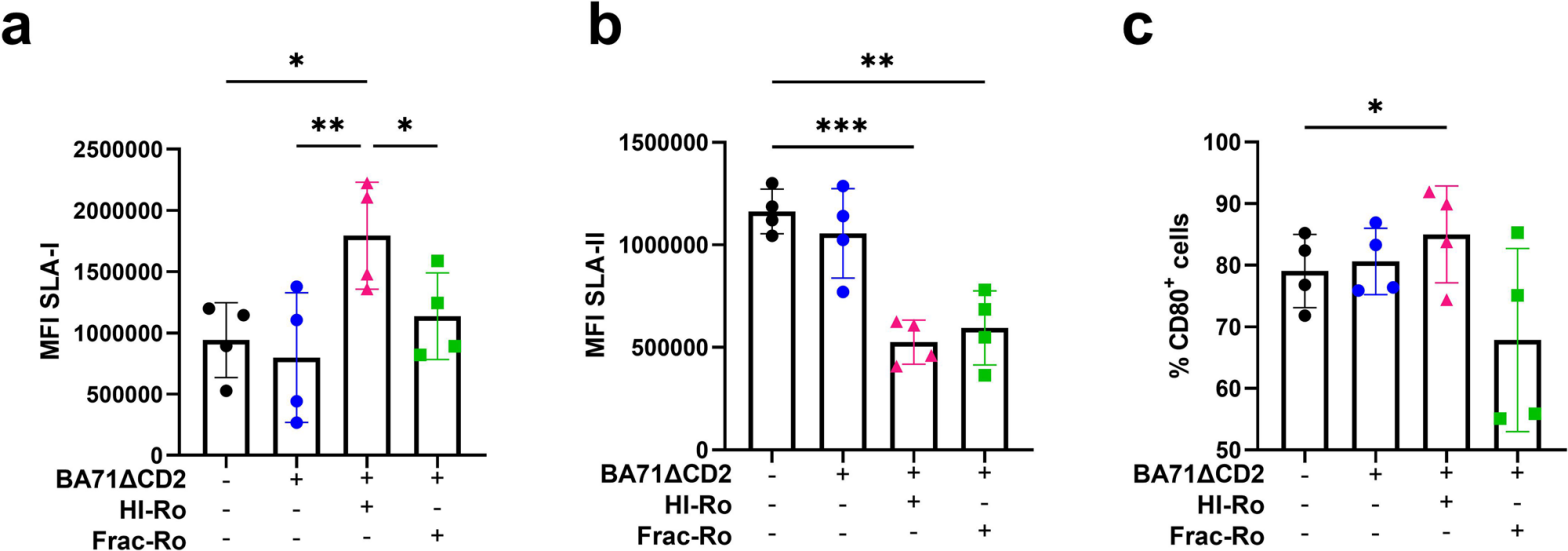
*Rothia*-derived compounds modulate antigen-presenting markers in macrophages. **a**–**c** Porcine alveolar macrophages were infected with the vaccine prototype BA71ΔCD2 for 2 hours at MOI 0.1, and subsequently treated with HI-Ro (MOI 50), Frac-Ro, or left untreated. At 72 h post-infection, the mean fluorescent intensity of surface SLA-I (**a**) and SLA-II (**b**), as well as the frequencies of CD80^+^ cells (**c**), were quantified by flow cytometry. Significant differences were determined using one-way ANOVA (*p-value ≤ 0.05, **≤ 0.01, ***≤ 0.001).

**Fig. 4:**
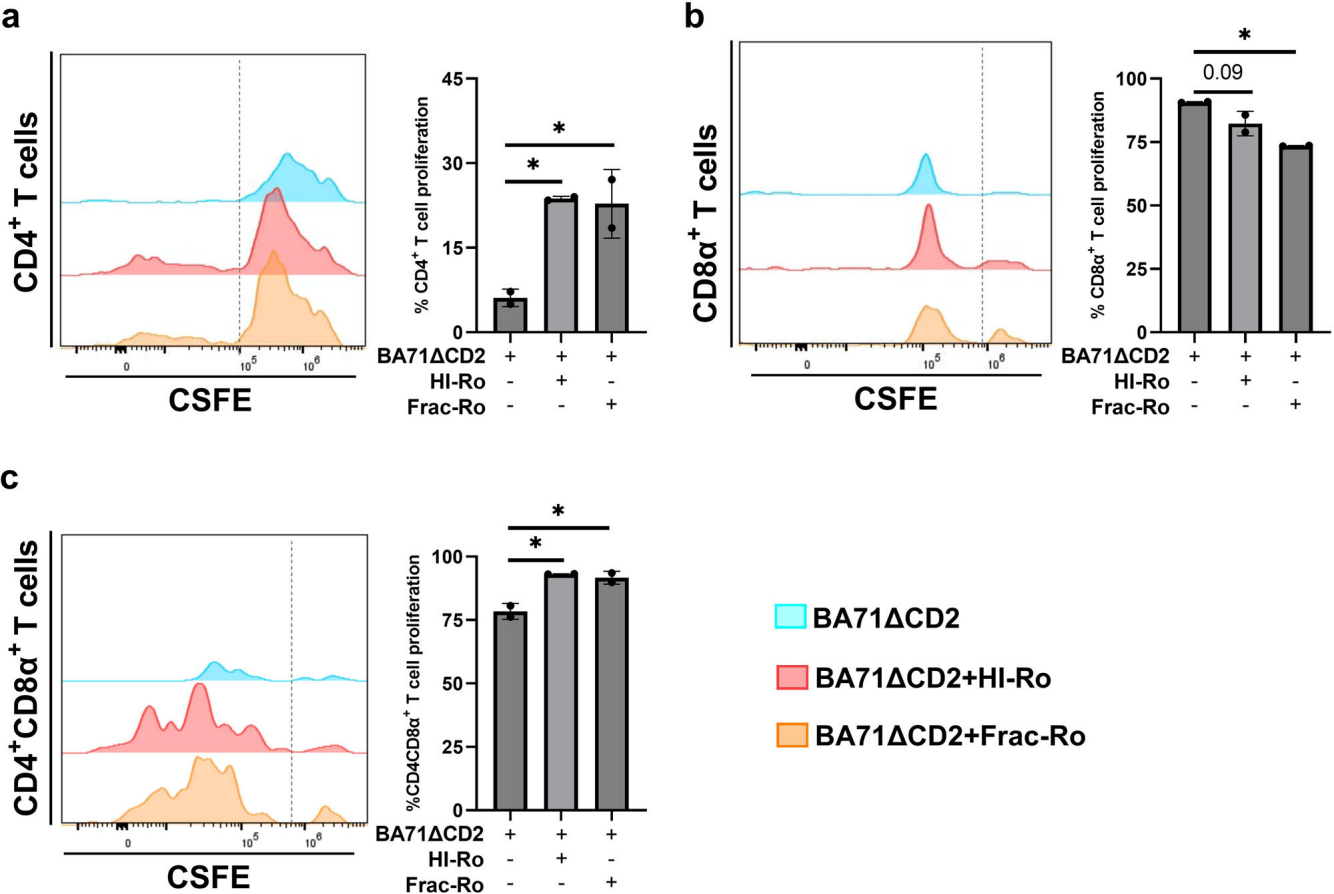
Supernatants from HI-Ro- and Frac-Ro-treated macrophages infected with BA71ΔCD2 enhance PHA-dependent T cell proliferation. **a**–**c** Porcine alveolar macrophages were infected with the vaccine prototype BA71ΔCD2 for 2 hours (h) at MOI 0.2, and subsequently treated with HI-Ro (MOI 50), Frac-Ro, or left untreated. At 24 h post-infection, culture supernatants were collected, filtered to remove virus, and used to stimulate CFSE-labelled peripheral blood mononuclear cells (PBMCs) together with a suboptimal PHA concentration (1 μg/ml). After five days, the frequencies of proliferating CD4^+^ (**a**), CD8α^+^ (**b**) and CD4^+^CD8α^+^ (**c**) T cells were quantified by flow cytometry. Significant differences were determined using one-way ANOVA (*p-value ≤ 0.05).

Next, we evaluated whether HI-Ro or Frac-Ro treatment of macrophages infected with the ASFV vaccine prototype BA71ΔCD2 can overcome the viral-induced immunosuppressive status, thus enhancing their functionality as antigen-presenting cells. BA71ΔCD2-infected PAMs were stimulated with HI-Ro or Frac-Ro, and compared to IFN-γ or LPS, and their responsiveness was analysed by cytokine production and transcriptomic analysis. As previously demonstrated in macrophages infected with the highly virulent ASFV strain Georgia2007/1^32^, BA71ΔCD2 infection likewise failed to induce cytokine production in PAMs. However, this lack of responsiveness was overcome in HI-Ro and LPS-treated cells, which robustly secreted TNF. Stimulation with Frac-Ro and IFN-γ showed a similar but not statistically significant tendency (Fig. 2a). In contrast, IFN-γ production was only induced in BA71ΔCD2-infected cells following Frac-Ro stimulation, showing similar results with IFN-γ, but not statistically significant (Fig. 2b). To note, IL-1β was secreted only in HI-Ro- and LPS-treated macrophages although this effect did not reach statistical significance (Fig. 2c). Finally, the expression levels of 24 genes related to innate immunity were upregulated upon HI-Ro-stimulation of BA71ΔCD2-infected PAMs compared with non-treated and infected or non-infected cells (Fig. 2d; Supplementary Data 1). In contrast, Frac-Ro only significantly increased the expression of a few genes, such as *CCL3*, *CCL4* and *TNF*. Beyond their higher activation status, *Rothia*-treated macrophages also displayed modulation of antigen-presentation markers. HI-Ro-stimulation increased surface SLA-I mean fluorescence intensity (MFI) and the frequency of CD80⁺ cells, whereas both *Rothia*-derived products reduced surface SLA-II MFI (Fig. 3). Together with the increase in SLA-I and CD80, the modulation of surface SLA-II supports a remodelling of the antigen-presentation pathways in *Rothia*-stimulated macrophages. Additionally, supernatants from HI-Ro- and Frac-Ro-stimulated macrophages enhanced PHA-dependent proliferation of CD4⁺ and CD4⁺CD8⁺ T cells (Fig. 4a, c). Taken together, these results indicate that the immunosuppressive state induced in ASFV-infected macrophages can be at least partially rescued by macrophage-activating components from *R. nasimurium*, suggesting their potential as adjuvants for ASFV-based LAVs.

### The use of HI-Ro or Frac-Ro as adjuvants impairs BA71ΔCD2-induced immunogenicity in pigs

**Fig. 5:**
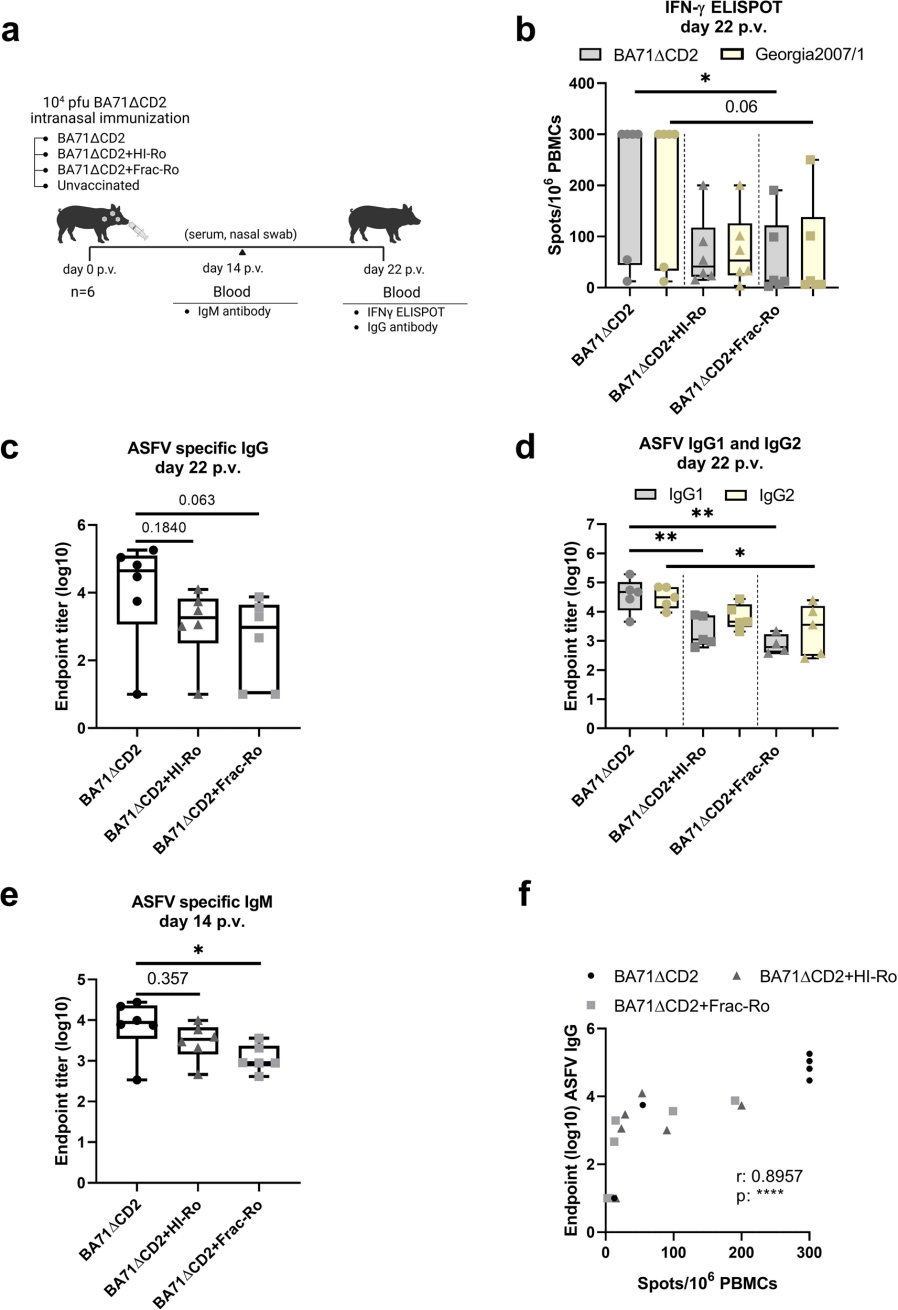
Combination of intranasal BA71ΔCD2 vaccination with HI-Ro or Frac-Ro impairs the induction of ASFV-specific cellular and humoral responses. **a** Schematic representation of the experimental design. Created with BioRender.com. **b** IFN-γ-producing cells in peripheral blood mononuclear cells (PBMCs) obtained at day 22 post-vaccination were quantified by ELISpot. Cells were stimulated in vitro with the ASFV-strains BA71ΔCD2 or Georgia2007/1. ASFV-specific IgG (**c**), IgG1, IgG2 (**d**), and IgM (**e**) in serum at the indicated time-points were titrated by ELISA. **f** Spearman correlation analysis between the number of ASFV-specific IFN-γ-producing cells and the ASFV-specific IgG titre for each animal. Significant differences were determined using one-way ANOVA (**b**–**e**) or Spearman correlation (**f**) (*p-value ≤ 0.05, ** ≤0.01, ****≤0.0001).

Intranasal vaccination with BA71ΔCD2 confers total or partial protection against a lethal challenge, depending on the vaccine dose^31^. Thus, to evaluate the adjuvant potential of HI-Ro or Frac-Ro, we combined each of them with a suboptimal single dose of BA71ΔCD2 (10^4^ pfu) (Fig. 5a). The use of adjuvants did not modify the safety profile of the BA71ΔCD2 vaccine prototype. Indeed, all vaccinated pigs showed a lack of clinical signs, regardless of the use of adjuvants, with rectal temperatures below 41 °C and low clinical scores as in unvaccinated pigs (Supplementary Fig. 4a, b). Consistently, virus loads in serum and nasal swabs were negative except for one animal vaccinated without adjuvant, showing low levels of virus (Supplementary Fig. 4c, d).

To evaluate the effect of using HI-Ro or Frac-Ro as adjuvants, we next analysed ASFV-specific cellular and humoral immune responses induced post-vaccination (p.v.) (Fig. 5a). Importantly, Frac-Ro group showed significantly lower numbers of ASFV-specific IFN-γ-producing cells at day 22 p.v., whereas the HI-Ro group displayed a similar but not significant reduction (Fig. 5b). Regarding humoral responses, between-group differences in total ASFV-specific IgG titres at day 22 p.v. did not reach statistical significance (Fig. 5c**;** Supplementary Fig. 5a). Nevertheless, ASFV-specific IgG1 and IgG2 titres (Fig. 5d**;** Supplementary Fig. 5b-c), as well as IgM titres at 14 days p.v. (Fig. 5e**;** Supplementary Fig. 5d), tended to be lower in at least one of the adjuvant-treated groups, although the magnitude of these differences varied and some did not reach significance. Finally, correlation analysis between ASFV-specific IFN-γ-producing cells and IgG titres at day 22 p.v. demonstrated that pigs responding to vaccination concomitantly triggered both cellular and humoral responses, regardless of the vaccination group (Fig. 5f). Finally, we evaluated whether the observed lowered immune responses could be influenced by a potential antigenic interference between *Rothia*- and BA71ΔCD2-derived antigens. To this end, we analyzed CD4^+^, CD8α^+^, or CD4^+^CD8α^+^ T cells responses after in vitro stimulation of peripheral blood mononuclear cells (PBMCs; day 22 p.v.) with a *Rothia* lysate from pigs inoculated with HI-Ro. No *Rothia*-specific CD4^+^, CD8α^+^, or CD4^+^CD8α^+^ T cell responses were detected, as indicated by the absence of CD154 and TNF expression (Supplementary Fig. 6). Overall, these results indicate that the use of HI-Ro or Frac-Ro as BA71ΔCD2 adjuvants dampens vaccine-induced immunity, equally affecting both systemic cellular and humoral responses.

### A combination of BA71ΔCD2 with HI-Ro or Frac-Ro reduces vaccine efficacy against a lethal ASFV infection

**Fig. 6:**
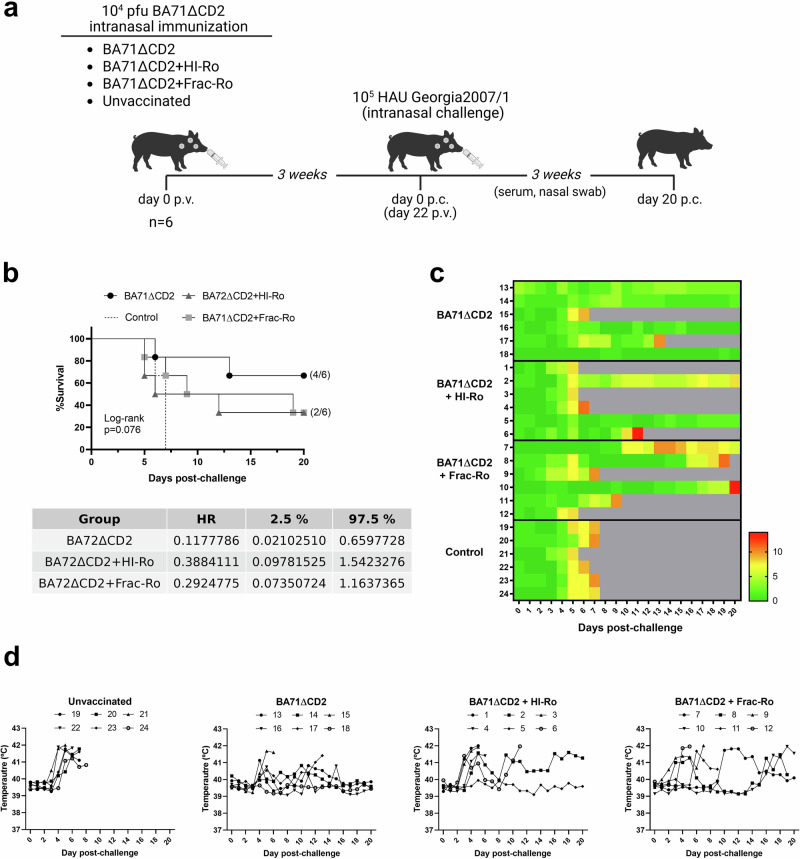
BA71ΔCD2 efficacy against a lethal ASFV challenge is reduced when combined with HI-Ro or Frac-Ro. **a** Graphical representation of the experimental design. Created with BioRender.com. **b** Survival plot showing the percentage of alive pigs at the indicated time points after intranasal challenge with Georgia2007/1. The number of surviving pigs at the end of the experiment is indicated in brackets. Hazard ratios (HR) and confidence intervals are shown in the table. Daily clinical scores (**c**) and rectal temperatures (**d**) of individual pigs after intranasal challenge. The severity of the clinical signs (score value) is represented by a coloured gradient: green (absence), yellow (mild), and orange/red (severe).

**Fig. 7:**
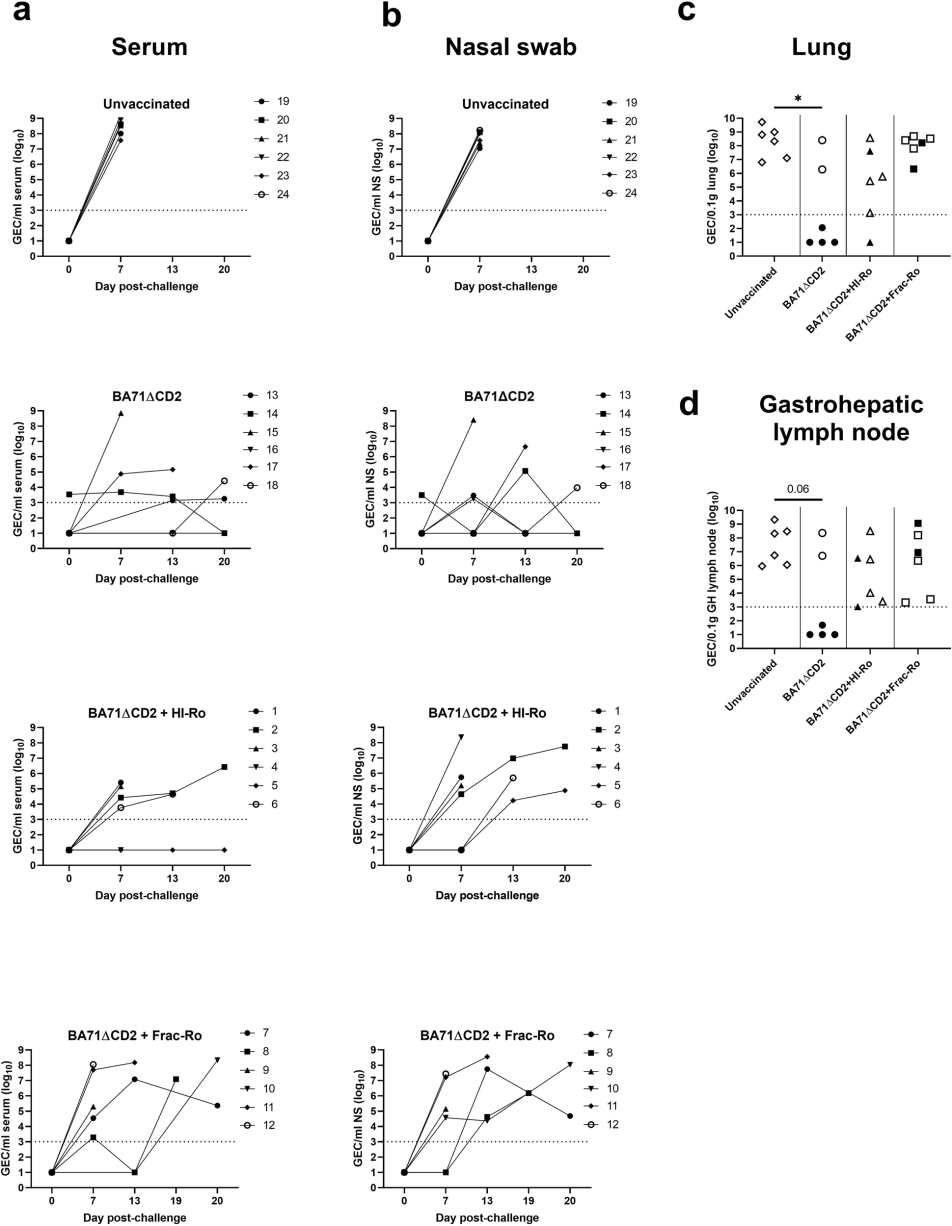
Pigs vaccinated with BA71ΔCD2 without adjuvants show lower viremia and virus shedding after lethal challenge. Virus loads in blood serum (**a**) and nasal swabs (**b**) at days 0, 7, 13, and 20 post-challenge measured by qPCR. Genomic equivalent copies (GEC) were quantified by the detection of the ASFV gene *PK*. Virus loads in the lung (**c**) and the gastrohepatic lymph node (**d**) on the day of sacrifice of each animal. Black-filled symbols represent animals surviving until the end of the study, and white symbols represent animals sacrificed before the end of the study. Significant differences were determined using one-way ANOVA (*p-value ≤ 0.05).

To evaluate the protection induced by BA71ΔCD2 combined with HI-Ro or Frac-Ro, we next challenged the pigs at 22 days p.v. with a lethal intranasal inoculation of the virulent Georgia2007/1 strain (Fig. 6a). As previously reported^31^, suboptimal vaccination with BA71ΔCD2 without HI-Ro or Frac-Ro conferred partial protection, with four out of six pigs (66.6%) surviving the lethal challenge, while all unvaccinated animals died between days 6-7 post-challenge (p.c.) (Fig. 6b), confirming the lethality of the inoculum. Importantly, in line with the lower immune responses induced when using HI-Ro or Frac-Ro (Fig. 5), pigs vaccinated with these adjuvants showed a notable drop in the vaccine efficacy. Indeed, only 2 out of 6 pigs (33.3%) from each group inoculated with HI-Ro or Frac-Ro survived the challenge (Fig. 6b). Moreover, only one of these four surviving pigs showed robust protection, while the other three pigs presented moderate to severe ASF clinical signs and temperatures exceeding 41 °C (Fig. 6c, d). The decline in the efficacy of adjuvanted vaccination groups was also reflected by uncontrolled viremia. Indeed, only one of the surviving pigs in the adjuvanted groups was negative until the end of the study (Fig. 7a**)**. Importantly, all pigs vaccinated with any of the two adjuvants, including the surviving ones, showed high levels of virus shedding in nasal swabs (Fig. 7b**)**. This uncontrolled virus replication was further validated analysing virus loads in lungs and gastrohepatic lymph nodes by qPCR, which were positive in almost all pigs vaccinated with adjuvant (Fig. 7c, d). These results were in contrast with the protection afforded in not adjuvanted BA71ΔCD2 vaccinated pigs, which did not show high systemic or local virus loads (Fig. 7a–d), demonstrating that HI-Ro or Frac-Ro hamper BA71ΔCD2-induced protective immunity.

### HI-Ro and Frac-Ro impair BA71ΔCD2 replication in porcine macrophages in vitro

**Fig. 8:**
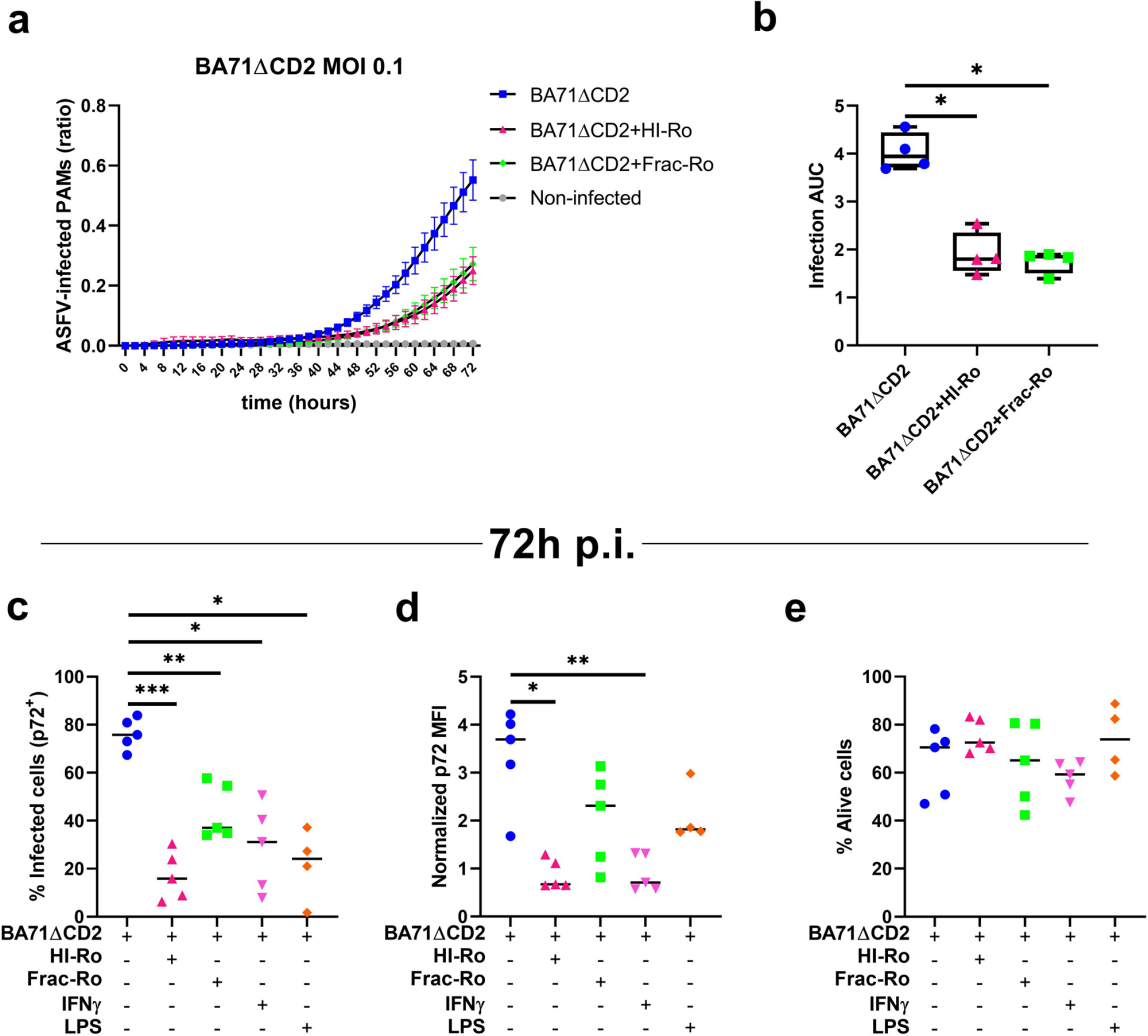
BA71ΔCD2 replication capability is reduced when combined with HI-Ro and Frac-Ro. **a**, **b** Porcine alveolar macrophages were infected with the fluorescently labelled BA71ΔCD2-mWasabi at MOI 0.1. Two hours (h) post-infection, cells were treated with HI-Ro (MOI 50), Frac-Ro or left untreated. **a** The percentages of BA71ΔCD2-infected cells were analysed every 2 h using time-lapse Incucyte analysis. **b** Area under the curve (AUC) for each condition obtained from the kinetics of percentages of infected cells. **c**–**e** BA71ΔCD2-infected PAMs (MOI 0.1) treated with HI-Ro (MOI 50), Frac-Ro, IFN-γ (0.1 μg/mL), LPS (10 μg/mL), or left untreated were analysed at 72 h post-infection by flow cytometry. The percentages of BA71ΔCD2-infected cells (**c**), the mean fluorescent intensity (MFI) of the p72 ASFV protein (**d**), and the percentage of alive cells (**e**) are shown. Significant differences were determined using a one-way ANOVA (*p-values ≤ 0.05, ** ≤0.01, *** ≤0.001).

**Fig. 9:**
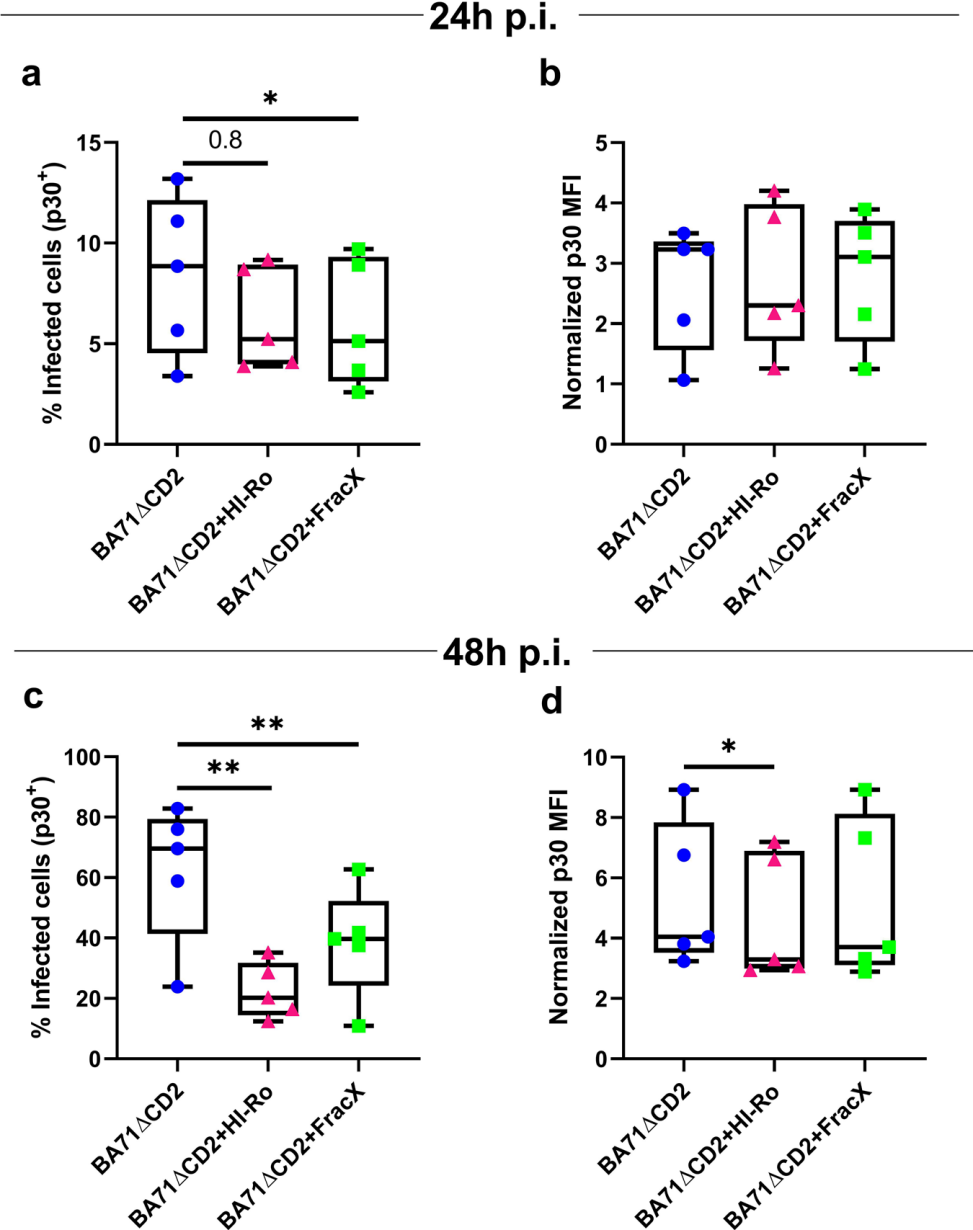
HI-Ro and Frac-Ro impair the early expression of the ASFV protein p30. **a**–**d** Porcine alveolar macrophages were infected with the BA71ΔCD2, and 2 hours (h) post-infection were treated with HI-Ro (MOI 50), Frac-Ro, or left untreated. At 24 (**a**, **b**) and 48 (**c**, **d**) h post-infection, the percentages of p30-positive infected cells (**a**, **c**) and the MFI of the early expressed ASFV protein p30 (**b**, **d**) were measured by flow cytometry. Significant differences were determined using a one-way ANOVA (*p*-values of *p-values ≤ 0.05, **≤0.01).

We have previously demonstrated that HI-Ro has anti-viral properties, reducing the replication capability of virulent ASFV and PRRSV strains in macrophages^32^. Based on the lower immune responses and suboptimal protection observed in pigs vaccinated with BA71ΔCD2 combined with HI-Ro or Frac-Ro, we hypothesized that these immunostimulants might similarly impair the replication of the LAV, thereby limiting antigen availability in vivo. To explore this hypothesis, we designed in vitro experiments to identify biological effects that could plausibly contribute to the reduced vaccine efficacy observed in vivo. First, we measured in vitro the replication kinetics of BA71ΔCD2 in PAMs treated or untreated with HI-Ro or Frac-Ro. Percentages of infected cells were quantified by time-lapse analysis of macrophages infected with a BA71ΔCD2 expressing a fluorescent fusion protein of the late viral structural protein p54 (BA71ΔCD2-mWasabi). Importantly, the presence of either HI-Ro or Frac-Ro resulted in a significant decrease in the replication capacity of the vaccine strain, regardless of the initial virus dose used (Fig. 8a, b**;** Supplementary Fig. 7a, b). These results were further validated by flow cytometry at 48- and 72- hours post-infection, analysing cells positive for the late-expressed viral protein p72. Indeed, both HI-Ro and Frac-Ro significantly decreased the percentage of infected cells (Fig. 8c**;** Supplementary Fig. 7c). This effect was probably mediated by the activation of macrophages, since similar results were obtained when infecting the cells together with stimulatory components IFN-γ or LPS (Fig. 8c**;** Supplementary Fig. 7c). Indeed, a short stimulation with HI-Ro or Frac-Ro prior to BA71ΔCD2 infection did not modify the replication capacity of the vaccine virus (Supplementary Fig. 8), thus further suggesting that a full activation of macrophages is required for the anti-viral effect. Importantly, also the mean fluorescent intensity of p72 was also decreased in BA71ΔCD2-infected and HI-Ro- or IFN-γ-treated cells, indicating the lower replicative capacity of the vaccine virus at the single cell level (Fig. 8d**;** Supplementary Fig. 7d). In this line, analysis of percentages of infected cells by the detection of the early viral protein p30 also demonstrated the lower replicative capacity of BA71ΔCD2 in the presence of HI-Ro or Frac-Ro (Fig. 9), denoting that the antiviral activity affects early steps of the virus replication cycle. Notably, these differences did not result from a cytotoxic effect of the treatments, since the percentages of alive cells were not significantly decreased in the presence of HI-Ro or Frac-Ro (Fig. 8e**;** Supplementary Fig. 7e). Together, these in vitro data demonstrate that both HI-Ro and Frac-Ro substantially impair the replication of the BA71ΔCD2 vaccine virus in fully activated macrophages, and provide a biologically plausible explanation for the reduced BA71ΔCD2-induced immunity and protection observed in pigs vaccinated with adjuvanted formulations.

## Discussion

While waiting for the development of efficient subunit vaccines against ASF, the use of ASFV-based LAVs with improved biosafety profiles might represent a temporary solution in affected areas^10^. Here, we have shown that any attempt to increase LAVs' biosafety by the use of bacterium-derived adjuvants should avoid the triggering of an antiviral state in macrophages. The use of immunostimulants such as HI-Ro and Frac-Ro might counteract the ASFV-mediated inhibition of macrophage functionality, and thus help in the induction of protective immune responses. However, inherent with this enhanced functional state of macrophages was the activation of an antiviral response that, as demonstrated in vitro, restricted the replication of the LAV. However, these mechanistic observations were limited to macrophage cultures, and thus their contribution to the in vivo phenotype must be considered as a plausible explanation rather than a demonstrated causal mechanism. Nevertheless, these results indicate that the use of adjuvants combined with ASFV-based LAVs must target specific immune components and/or signalling pathways promoting innate immune responses, while allowing enough LAV replication to reach the threshold of viral antigen levels required to trigger a protective adaptive immunity.

The use of LAVs is the only current vaccine strategy conferring solid protection against ASF, as evidenced by the two LAV ASF vaccines registered in Vietnam^7^. However, their implementation in the field is limited due to biosafety concerns^6,11^. Indeed, the balance between virus attenuation and immunogenicity is very subtle, with the consequent potential of reversion to virulence^5,8,33^. Nonetheless, despite these concerns, there is an open discussion on the potential benefits derived from a rational use of LAVs in areas affected by virulent ASFV strains^10^. Thus, the optimization of LAVs to improve their biosafety profile may contribute to controlling ASFV spread while diminishing the risk of adverse events. In this scenario, the use of adjuvants represents a potential strategy that deserves investigation. Adjuvants boost vaccine-induced immune responses while reducing the amount of antigen required for effective immunization^34^. Therefore, the combination of adjuvants with ASF LAVs might lead to a reduction of the vaccine dose with the subsequent drop in potential biosafety issues. However, vaccines based on live viruses have a robust endogenous stimulatory capability, and typically do not need adjuvants to trigger immune responses^25^. But this concept should be reconsidered for viruses suppressing innate immune signalling pathways to evade antiviral immunity. Indeed, here we have demonstrated that BA71ΔCD2 combined with HI-Ro or Frac-Ro rescues the functionality of infected macrophages, promoting cytokine production, the upregulation of activation markers, and the acquisition of antigen-presentation features. Indeed, *Rothia* stimulation induced a coordinated modulation of antigen-presentation pathways, characterized by increased SLA-I and CD80 expression together with dynamic regulation of SLA-II, consistent with enhanced antigen-presenting cell (APC) functionality. Importantly, transient regulation of surface MHC-II during APC activation has been associated with intracellular trafficking through peptide-loading compartments, a central feature of classical antigen-processing pathways^35,36^. These results confirm the feasibility of enhancing innate responses in ASF LAVs but do not indicate whether such enhancement improves or limits vaccine performance in vivo, as additional host-virus interaction mechanisms are likely involved. Another limitation of this work is that it focuses on a single LAV. However, the attenuation of ASFV is usually achieved by mutations or deletions in genes involved in immunosuppressive mechanisms^5,14^, such as the CD2v-encoding gene deleted from the BA71ΔCD2^37,38^. Although these modifications reduce virulence, the complexity of the virus prevents a complete loss of its capacity to compromise macrophage functionality^39–41^. Thus, it is likely that similar results obtained with the BA71ΔCD2 would be obtained using other ASFV attenuated strains. Notably, this adjuvant-mediated strategy could also be applied to LAVs against porcine reproductive and respiratory syndrome (PRRS) and classical swine fever (CSF) viruses, which also infect macrophages and suppress innate immunity^42,43^.

The enhanced functionality of BA71ΔCD2-infected macrophages observed in vitro upon both HI-Ro and Frac-Ro stimulation did not result in an improvement in vaccine efficacy against a lethal challenge in vivo, regardless of the adjuvant used. Instead, the protection afforded upon administration of the adjuvanted LAVs was significantly reduced. Despite the compositional differences of the two immunostimulants used in this study, HI-Ro and Frac-Ro similarly hampered vaccine performance in vivo. This aligns with our transcriptomic comparisons showing extensive overlap in the differentially expressed genes induced by both adjuvants, indicating that they converge on highly similar innate activation programmes. These shared effects highlight the need for deeper biochemical characterization to identify the specific *Rothia*-derived components responsible for this outcome. In this regard, our preliminary analyses indicate that Frac-Ro contains glycolipids compatible with the previously described as immunostimulatory compounds from *Rothia mucilaginosa*^44^.

Vaccine efficacy against any pathogen depends on several factors, but the antigen levels and the type and magnitude of the induced immune responses are particularly critical^45^. In this line, HI-Ro or Frac-Ro adjuvanted BA71ΔCD2 generated lower levels of ASFV-specific humoral and cellular responses than the non-adjuvanted LAV, but did not alter the quality of virus-specific immune responses. Indeed, the results indicate that HI-Ro and Frac-Ro neither significantly modified virus-specific antibody isotypes, nor the ratio between antibody and cellular responses induced in each pig, and did not promote competition between bacterial and viral antigens. Thus, it is likely that the decreased vaccine efficacy was linked to the insufficient induction of virus-specific antibodies and T cells, which did not reach the required thresholds to protect the animals. Mechanistically, it is reasonable to hypothesise that reduced replication of the BA71ΔCD2 LAV in vivo could contribute to this decreased immunogenicity. This hypothesis is supported by the lower capability of the BA71ΔCD2 to replicate in vitro in the presence of HI-Ro or Frac-Ro, but confirmation of this mechanism in vivo will require additional experiments. Moreover, future studies should evaluate in vivo dose–response relationships and the potential benefits of modulating the timing of adjuvant and LAV administration, as well as assessing whether alternative vaccination routes could influence early innate responses without excessively restricting viral replication. Indeed, ASFV is sensitive to interferon-induced responses, as shown both in vitro^40^ and in vivo^46^. Moreover, an elegant study in a mouse model showed that short-term IFN-I blockade during immunization with several viral vaccines enhances their efficacy through a transient “spike” in antigen levels^47^. Thus, the capability of *Rothia*-derived components to induce the expression of interferon-stimulated genes (ISGs) in BA71ΔCD2-infected macrophages might restrain the virus replication rate with the consequent decrease of antigen production. However, given the complexity of vaccine-induced immunity in vivo, additional mechanisms may also contribute, and our in vitro model does not capture the full diversity of cell types and tissues involved. This complexity is evidenced by studies performed using adjuvants for live attenuated influenza virus vaccines. In mice, α-C-GalCer increased influenza-specific humoral and cellular responses in a Natural killer T cell-dependent manner, and subsequently reduced both morbidity and mortality after challenge^29^. However, in line with our results, α-C-GalCer impaired the replication of the live vaccine in pigs, reducing vaccine-induced cross-protective immune responses^30^. These findings further illustrate that adjuvant effects on LAV replication and efficacy can be species-specific and context-dependent, reinforcing the need for targeted mechanistic studies.

The results discussed above clearly indicate that a fine-tuned and rational selection of adjuvants is necessary to exploit their use for ASF LAVs. Thus, to obtain an adjuvanted vaccine formulation with higher biosafety profiles, further research is needed to better characterize the innate immune responses associated with a favourable outcome upon ASFV or LAV infections. In this context, several early correlates of protection have been recently identified upon immunization with the incompletely attenuated ASFV strain Estonia 2014^48^. In this model, the prompt activation of antigen presenting cells (APC), plasma cells, and T cells correlated with the induction of protective immunity. However, a systemic antiviral response and high levels of IFNα in serum were also identified as favourable parameters. These results contrast with our association of an antiviral response in macrophages with a decreased vaccine efficacy. However, this study analysed systemic responses in blood in a model characterised by long viremia, and infection outcomes are usually linked to early events occurring at the site of initial viral replication^49–51^. This is especially important for completely attenuated ASFV strains such as BA71ΔCD2 and other LAVs, which usually do not spread systemically or have short viraemic periods^5^. Thus, further studies should focus on identifying early local correlates of protection and clarifying how innate responses at the site of replication influence the balance between LAV antigen production and antiviral restriction. Rationally selected adjuvants should achieve a good balance between an enhanced immune functionality in innate immune cells, and the capability of the vaccine virus to replicate and produce the required antigen levels to trigger a protective adaptive immune response.

## Methods

### Preparation of heat-inactivated *R. nasimurium* (HI-Ro) and cell-free supernatant (Frac-Ro)

Growth of *R. nasimurium* to obtain the required multiplicity of infection (MOI) was performed as previously described^32^. The bacterium was quantified by serial dilutions and plating, and inactivated at 65 °C for 1 hour. Inactivation was confirmed by the absence of overnight growth on chocolate agar at 37°C with 5% of CO_2_. To obtain the cell-free supernatant, a plate with a *R. nasimurium* overnight culture was washed using 10 mL of phosphate-buffered saline (PBS), and centrifuged at 2.500 x *g* 10 min. The cell-free resulting supernatant was sterilized using a 0.2 μm filter. The different fractions from the supernatant were obtained using centrifugal filters with a molecular weight cut-off (MWCO) of 100 or 10 kDa (Amicon UFC9100, UFC9010). Supernatant fractions of ≥100, 100-10, and ≤10 MWCO kDa were recovered with 10 mL of Roswell Park Memorial Institute (RPMI) 1640 medium (Gibco). All *R. nasimurium*–derived adjuvant batches were produced following a standardized protocol, and each new batch underwent an in vitro quality-control test before use. Specifically, we assessed its ability to stimulate PAMs and verified that TNF induction always fell within a similar range. Only batches meeting these criteria were used in the study. For in vitro stimulatory assays, HI-Ro was first centrifuged at 2500 x *g* 10 min and resuspended in the corresponding media at MOI 50. Frac-Ro was diluted 1:2 in the corresponding x2 concentrated media. LPS (InvivoGen; tlrl-eblps) and IFN-γ (KingFisher) were used at 10 μg/ml and 0.1 μg/mL, respectively, as previously described^32,52^. For in vivo experiments, HI-Ro was used at 10^7^ cfu/mL, and Frac-Ro was diluted 1:2 with PBS. Because Frac-Ro and derived cell-free fractions do not contain inactivated bacteria, their concentration cannot be expressed as a multiplicity of infection (MOI). Therefore, doses of Frac-Ro used in in vitro assays are reported as dilution factors (v/v), which reflect the maximum concentration obtainable from the extraction procedure. No direct MOI-based comparison with HI-Ro is possible due to their fundamentally different nature and concentration units.

### Viruses

The virulent ASFV strain Georgia2007/1 (genotype II) was kindly provided by Dr. Linda Dixon (WOAH reference laboratory, Pirbright Institute, UK), grown in PAMs, and titrated by hemadsorption assay^53^. The BA71ΔCD2 is a live attenuated strain lacking the CD2v gene (EP402R) obtained by homologous recombination from the parental virulent BA71 strain^54^. It was grown in the stabilised COS-1 cell line (ATCC), and titrated by immunoperoxidase monolayer assay (IPMA)^54^. Titres were calculated by the Reed and Muench method, and expressed as 50% haemagglutination activity units (HAU_50_)/mL or 50% tissue culture infectious dose. TCID_50_ titres obtained from IPMA were converted to plaque-forming units (pfu) by applying the Poisson distribution (pfu/mL ≈ 0.7 × TCID_50_/mL).

### Porcine alveolar macrophages and cytokine quantification from cell culture supernatants

Porcine alveolar macrophages (PAMs) were obtained through lung lavage of healthy animals (from Landrace, Landrace x Duroc, and Landrace x Large White pig breeds) as previously described^32^. Cells were maintained in Roswell Park Memorial Institute (RPMI) 1640 medium (Gibco) supplemented with 10% heat-inactivated foetal calf serum (FCS) (Invitrogen), 1% of penicillin-streptomycin/mL (P/S) (Invitrogen), 1% of L-glutamine (Invitrogen), and 0.5% of nystatin (Invitrogen). For in vitro stimulation assays, three to four lots of PAMs were seeded in 48-well flat-bottom plates at 5–6 × 10^5^ cells/well and left overnight at 37 °C. Then, cells were incubated with live *R. nasimurium*, HI-Ro, or Frac-Ro at the indicated concentrations and time points. After stimulation, supernatants were collected and stored at -80°C for TNF (R&D system), IL-1β (R&D system), and IFN-γ (King Fisher) quantification by ELISA, following the manufacturer's procedure.

### RNA-seq library preparation and sequencing

Four lots of PAMs were seeded at 5 × 10^6^ cells/well in a 6-well flat-bottom plate, and left overnight at 37 °C with 5% of CO_2_. PAMs were stimulated for 6 or 24 hours with Frac-Ro (dilution 1:2). Non-stimulated cells were used as a control. Total RNA was isolated using the RNeasy Mini Kit (Qiagen) following the manufacturer’s protocol. To ensure RNA quality, DNase I treatment was performed for 15 minutes at room temperature. RNA was sequenced at the Centre Nacional d’Anàlisi Genòmica (CNAG), Barcelona, Spain. Total RNA concentration was quantified using Qubit RNA BR Assay kit (ThermoFisher Scientific), and the RNA integrity was estimated by Agilent Bioanalyzer. The RNASeq libraries were prepared with KAPA mRNA HyperPrep Kit (Roche) following the manufacturer’s recommendations, starting with 500 ng of total RNA as the input material. The library was quality controlled on an Agilent 2100 Bioanalyzer with the DNA 7500 assay. The libraries were sequenced on NovaSeq 6000 (Illumina) with a read length of 2 x 51bp, following the manufacturer’s protocol for dual indexing. Image analysis, base callin,g and quality scoring of the run were processed using the manufacturer’s software Real Time Analysis.

### RNA-seq bioinformatic analysis

Illumina reads were aligned to the *Sus scrofa* reference genome (Sscrofa11.1) with STAR (v2.7.8a) using ENCODE-recommended parameters^55^. Gene-level quantification used RSEM (v1.3.0) against Ensembl Sus scrofa annotation release 110^56^. Differential expression was tested with limma (v3.42.3). TMM was applied to compute library-size/compositional scaling factors, and voom converted counts to log2-counts-per-million (logCPM) while modelling the mean–variance trend on logCPM to derive observation-level precision weights^57,58^. Linear models were fit to the weighted logCPM values. Given the paired design, inter-individual effects were modelled with duplicateCorrelation; we extracted pairwise contrasts and the treatment × vaccination interaction. Genes were called DE at Benjamini–Hochberg FDR < 0.05. Functional enrichment used DAVID (http://david.ncifcrf.gov/)^59^.

### Animal experiment

All animal experiments were conducted in accordance with the European Directive 2010/63/EU for animal experiments and the Spanish legislation (RD 53/2013) on the protection of animals used for scientific purposes. Experimental procedures were approved by the Ethics Committee on Animal Experimentation of the *Generalitat de Catalunya* (Comitè Ètic d’Experimentació Animal de la Generalitat de Catalunya; protocol code 12121, approved on 2024/10/09).

All in vivo studies were performed in the biosafety level 3 facilities at *Centre de Recerca en Sanitat Animal* (IRTA-CReSA, Barcelona). Domestic pigs (Landrace x Large White), males, were housed in groups under controlled environmental conditions with ad libitum access to food and water and were allowed a 7-day acclimatization period prior to the start of the experiments. Pigs were randomly divided into four groups of six animals, and vaccinated at seven weeks of age. Three different vaccine formulations were tested: non-adjuvanted vaccine, HI-Ro-adjuvanted vaccine [10^7^ cfu/mL], and Frac-Ro-adjuvanted vaccine [dilution 1:/2]. In all cases, vaccinated animals were intranasally inoculated with 10^4^ pfu/animal of the attenuated LAV prototype BA71ΔCD2. Each animal received 2 mL of the vaccine diluted in PBS via intranasal inoculation (1 mL/nostril). The remaining six animals were used as unvaccinated controls and received 2 mL of PBS alone (1 mL/nostril). The intranasal route was selected because BA71ΔCD2 has previously shown safety and protective efficacy via this route, and it mimics the natural oronasal exposure of ASFV^31^. At day 22 post-vaccination (p.v.), all animals received 2 mL of an intranasal challenge containing 10^5^ HAU_50_ of the highly virulent ASFV strain Georgia2007/1 (1 mL/nostril). Rectal temperature and clinical signs were monitored daily during all the experiments as previously described^60^. Sera and nasal swabs were taken at 0, 7, 15, and 22 days p.v., and 0, 7, 13, and 20 days post-challenge (p.c.) to quantify viral loads by qPCR and ASFV-specific antibodies by ELISA. PBMCs were isolated at day 22 p.v. (day 0 p.c.) to measure ASFV-specific IFN-γ-producing cells by ELISpot.

Humane endpoints were applied according to institutional guidelines. Animals showing severe clinical signs compatible with advanced ASF, including prostration, severe respiratory distress, ataxia, spasticity, paralysis, or convulsions, were immediately euthanized to minimize suffering. In addition, animals reaching a clinical score greater than 12 according to the predefined clinical scoring system were euthanized. In cases where moderate clinical signs compatible with ASF were detected, animals were also evaluated for humane endpoint application in order to avoid unnecessary suffering once infection had been clearly established. Euthanasia was performed by intravenous administration of sodium pentobarbital (140 mg/kg; 200 mg/mL) by trained veterinary staff. This method induces rapid loss of consciousness followed by cardiac arrest. Death was confirmed by the permanent cessation of circulation.

No animals were excluded from the analysis. Animal facility veterinarians responsible for monitoring animal welfare were blinded to group allocations during the experiments; however, investigators were not blinded during data collection and analysis. On the last day of the study, lung and gastrohepatic lymph node tissues from each animal were isolated and kept at −80 °C until analysis.

### Quantitative PCR for the detection of ASFV

ASFV loads in sera, nasal swabs, and tissues were assessed by SYBR Green qPCR targeting the ASFV serine protein kinase gene (R298L; PK) as previously described^54^. Briefly, the viral genomic DNA was obtained using IndiMag® Pathogen Kit (INDICAL Bioscience) in a semi-automated manner by using a KingFisher System (Thermo) according to the manufacturer’s instructions. qPCR amplifications were performed in duplicates using the corresponding standards for absolute quantification. The results were expressed as log_10_ genome-equivalent copies (GEC) per millilitre of sera or nasal swab, or per 0.1 g of tissue. The detection limit of the technique was 10^3^ GEC/mL.

### Enzyme-linked immunosorbent assay (ELISA)

ASFV-specific antibodies in pig sera were detected by the WOAH-approved ELISA based on soluble extracts from ASFV-infected cells^61^. Samples were serially diluted from 1/100 to 1/1.562.500 to calculate the endpoint-titration, with a cut-off defined as the average negative control plus three times the standard deviation of the negative control. Positive sera were detected using the secondary peroxidase-conjugate antibodies: rabbit anti-pig IgG at 1/20,000 dilution (Sigma-Aldrich), anti-pig IgG1 at 1/1000 (Bio-Rad), anti-pig IgG2 at 1/1000 (Bio-Rad), or anti-pig IgM at 1/100,000 (BioRad). Soluble 3,3’,5,5’-tetramethylbenzidine (TMB, Sigma-Aldrich) was used as a specific peroxidase substrate. H_2_SO_4_ at 1 N was used as a stop solution, and plates were read at 450 nm. All samples were run in technical duplicates.

### IFN-γ enzyme-linked immunosorbent spot (ELISpot) assay

The IFN-γ-ELISpot assay was performed as previously described^62^. Peripheral blood mononuclear cells (PBMC) were separated from whole blood by density-gradient centrifugation with Histopaque 1077 (Sigma). Next, IFN-γ-secreting cells in PBMCs were measured by ELISpot assay using the purified mouse anti-pig IFN-γ (clone P2G10, BD Pharmingen) as capture antibody and biotinylated mouse anti-porcine IFN-γ antibody (clone P2C11, BD Pharmingen) as detection antibody. PBMCs were stimulated with BA71ΔCD2 or Georgia2007/1 at a MOI of 0.2, and incubated for 16 hours at 37 ˚ C, 5% CO_2_. Sample scoring ≥300 spots/million PBMCs received a score of 300. Samples approaching this ceiling were not diluted further, so values at the cap may underestimate true spot counts.

### Luminex-based multiplex assay

PAMs were stimulated for 24 hours with the three cell-free supernatant fractions, and supernatants were recovered and kept at −80 °C until analysis. Cytokine levels were quantified using the Luminex xMAP technology following the manufacturer’s instructions. Measurements included IFN-α, IL-1β, IL-4, IL-6, IL-12, and IL-10, IL-12p40 (ProcartaPlex Porcine Cytokine & Chemokine Panel 1; Thermo-Fisher Scientific). Concentrations of each cytokine were calculated using the xPONENT software (Luminex).

### Microfluidic quantitative PCR assay

Five lots of PAMs were seeded at 6 × 10^5^ cells/well in a 48-well flat-bottom plate and left overnight at 37 °C with 5% of CO_2_. Cells were infected for 2 h with the BA71ΔCD2 ASFV strain at MOI 0.1 in 100 μl/well of RPMI supplemented with 1% L-glutamine. After infection, cells were stimulated overnight with HI-Ro or Frac-Ro. Total RNA was isolated using the RNeasy Mini Kit (Qiagen) following the manufacturer’s protocol. Concentration was quantified using the Qubit RNA BR Assay kit (ThermoFisher Scientific), and the RNA integrity was estimated by Agilent Bioanalyzer. cDNA was obtained from 150 ng of total RNA using the PrimeScript RT reagent Kit (Takara, Japan) following the manufacturer’s instructions. Primer design (Supplementary Data 2) and validation were performed as previously described^63^. Gene expression levels were measured in duplicates using a microfluidic qPCR with the 48.48 Dynamic Array integrated fluidic circuit of the Biomark HD system (Fluidigm Corporation). Data was analyzed applying the relative standard curve method and using the Fluidigm Real-Time PCR analysis software 4.1.3 and the DAG expression software 1.0.5.6^64^. Target gene expression levels were normalized against the average of three reference control genes (YWHAZ, RPL4, and GAPDH), and z-score normalized values were represented in a Heatmap.

### Time-lapse microscopy of viral infection kinetics

The BA71ΔCD2-mWasabi fluorescent virus, encoding the mWasabi fluorescent protein (https://www.fpbase.org/protein/mwasabi/) fused to the C-terminus of the p54 ASFV protein, was generated by CRISPR technology as previously described^32^. To evaluate the capability of BA71ΔCD2 to replicate in the presence of HI-Ro and Frac-Ro, PAMs were seeded at 1.5×10^5^ cells/well and infected with the BA71ΔCD2-mWasabi at MOI 0.1 or 3.5 in RPMI supplemented with 1% L-glutamine for 2 hours. Next, cells were treated with HI-Ro or Frac-Ro. Viral infection kinetics were then quantified over 72 hours by time-lapse microscopy using an IncuCyte® SX5 (Sartorius BioAnalytical Instruments Inc, CA, USA). Specifically, plates were imaged every 2 h at 20x (4 fields of view per well) using the device’s “AI Scan” module in two channels (phase and green) using default acquisition parameters (green acquisition time 300 ms). Cell detection was performed using the “AI Cell Health” module (Segmentation Sensitivity = 0.6) without filtering for cell size. Classification of detected cells across the dimensions “uninfected/infected” was finally performed using the “Cell-by-Cell Classification” module: infected cells were identified based on a Green Mean Intensity threshold of 0.6 GCU (set empirically to have ~99% of cells in uninfected controls shown as negative).

### Flow cytometry

PAMs were seeded at 6 × 10^5^ cells/well in a 48-well flat-bottom plate infected with BA71ΔCD2 at MOI 0.1 for 2 hours in 100 μl/well of RPMI supplemented with 1% L-glutamine. Next, cells were treated with HI-Ro, Frac-Ro, or left untreated. Stored PBMCs from day 0 post-challenge were thawed and seeded in 96-well flat-bottom plates and stimulated with *Rothia* lysate (10 μg/mL) or with superantigen staphylococcal enterotoxin B (SEB; 2 ug/mL). For TNF detection, PBMCs were stimulated 6 hours plus 2 hours with Brefeldin A (BD GolgiPlug protein transport inhibitor) at 37˚C. At indicated time points post-infection or post-stimulation, cells were stained for viability with LIVE/DEAD Fixable Violet Dead or LIVE/DEAD Fixable Red Kit following the manufacturer’s instructions (ThermoFisher). Blockage of Fc receptors was performed in PBS containing 5% of porcine serum (Gibco) for 15 min on ice prior to antibody staining. For extracellular staining of PAMs, cells were incubated with anti-SLA-II (Bio-Rad; 2E9/13), anti-SLA-I (Bio-Rad; JM1E3), and anti-CD80 (Invitrogen; 16-10A1). For extracellular staining of PBMCs, the antibodies anti-CD3 (BD Biosciences; BB23-8E6-8C8), anti-CD-CD4 (BD Biosciences; 74-12-4), anti-CD8a (BD Biosciences; 76-2-11), and anti-CD153 (eBioscience; 5C8) were used. For intracellular staining of virus-infected PAMs, cells were fixed and permeabilized with the BD Cytofix/Cytoperm Kit (BD Biosciences) according to the manufacturer’s protocol, and incubated for 30 min on ice in Perm/Wash buffer with anti-p72 antibody (Eurofins Ingenasa; 1BC11) or anti-p30 antibody (hybridoma 1E12F5H2) kindly provided by M. Domínguez for ASFV. Secondary antibodies used were anti-mouse IgG1 (eBioscience; M1-14D12) and anti-mouse IgG2a (ThermoFisher; AF568), respectively. For intracellular TNF detection in PBMCs, cells were fixed, permeabilized, and stained with anti-TNF (BioLegend; MAb11). For the proliferation assay, PAMs were infected with BA71ΔCD2 at an MOI 0.2 for 2 h, followed by stimulation with HI‑Ro, Frac‑Ro, or left untreated. After 24 hours, supernatants were collected and filtered using a centrifugal filter with a MWCO of 100 KDa to remove virus particles. PBMCs were labelled with CFSE (ThermoFisher) at 5 μM and stimulated with the filtrated supernatants in combination with suboptimal PHA (1 μg/ml). After 5 days, proliferation of CD4^+^, CD8α^+^, and CD4^+^CD8α^+^ T cells was determined as previously described. In all cases, samples were acquired in a BD FACSAria IIu flow cytometer (BD Biosciences), and data were analysed using FlowJo v10.8.1 software (Tree Star Inc).

### Molecular profiling of Frac-Ro

High-resolution UHPLC-QTOF mass spectrometry with electrospray ionization (ESI) in positive mode was performed to characterize the molecular composition of the bacterial culture supernatant fraction used as adjuvant (Frac-Ro). The sample was filtered through a 0.2 μm Nylon membrane prior to injection. Initial characterization was conducted by direct infusion TOF-MS acquisition over an m/z range of 100–2500 Da. Subsequently, chromatographic separation on a BEH C18 1,7 µm (100 × 2.1 mm) column was coupled to SWATH® acquisition to obtain both MS and MS/MS spectra. Mass assignments were based on sodium adducts ([M+Na]⁺), and theoretical and experimental masses were compared to assess mass accuracy and molecular consistency.

### Statistical analyses

Graphics were created and analysed using the Prism version 8.0.2 software (GraphPad), and RStudio. Each statistical test is indicated in the corresponding figure legend. Statistical difference was set up at: ns *p* > 0.05; *p $\le$ 0.05; **p $\le$ 0.01; ***p $\le$ 0.001; ****p $\le$ 0.0001.

## Supplementary information

Supplementary Information

## Data Availability

The datasets generated for this study are available in the NCBI Gene Expression Omnibus (GEO) under the following accession number: GSE288520 (https://www.ncbi.nlm.nih.gov/geo/).

## Code availability

No custom code was used for data processing or analysis in this study. All analyses were performed using standard, publicly available software as described in the Methods section.
