# Supplementary material for: Adjuvant-induced macrophage activation compromises BA71ΔCD2-mediated protection against African swine fever virus
Source: NPJ Vaccines. 2026 Apr 24;11:141. doi: 10.1038/s41541-026-01461-5 (PMC13346925; doi:10.1038/s41541-026-01461-5)
Supplement: Supplementary file 1 — Supplementary Information [file 41541_2026_1461_MOESM1_ESM.pdf]

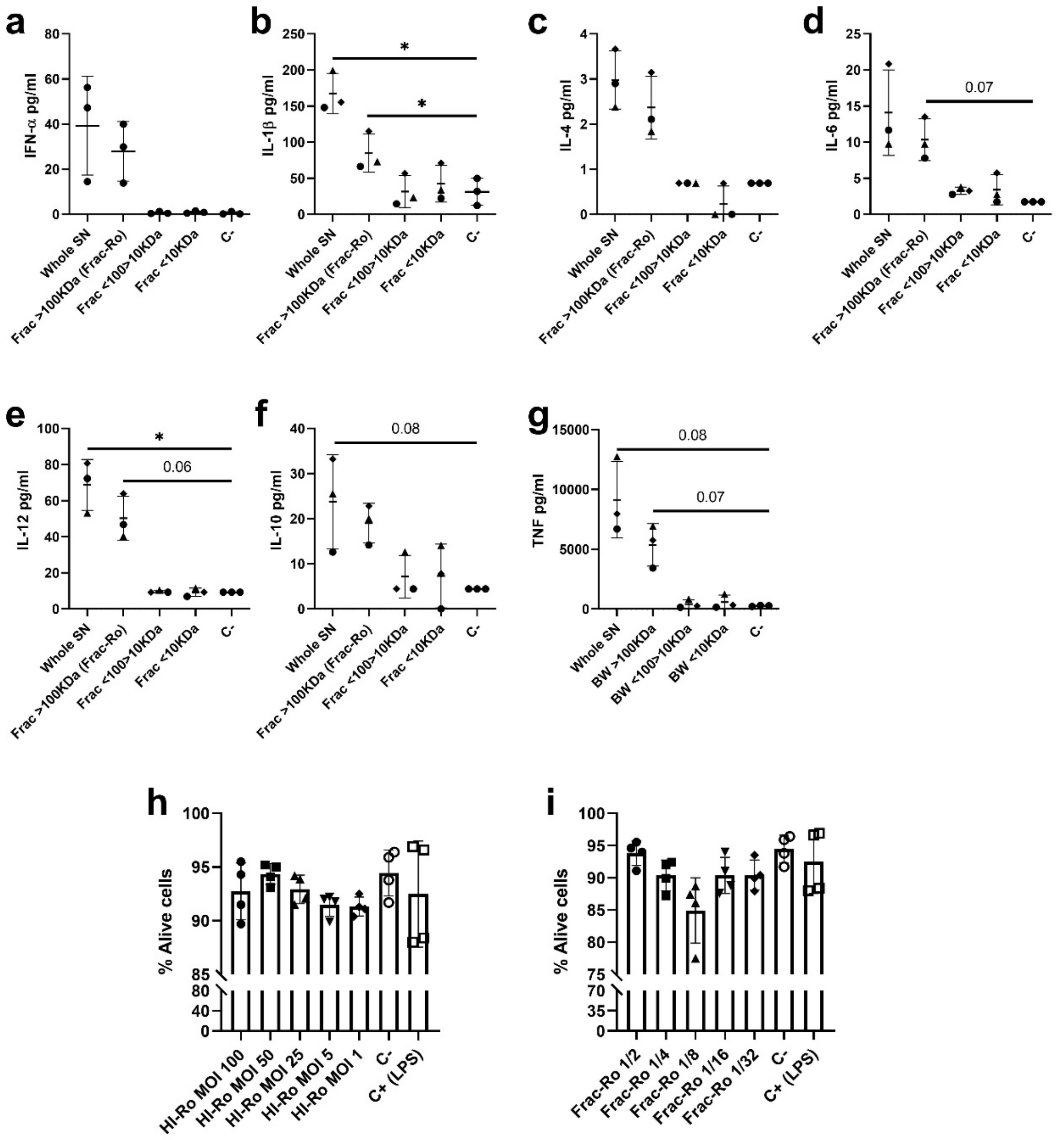

Supplementary Figure 1. PAMs were stimulated for 24 hours (h) with the different supernatant fractions separated by molecular weights. Levels of IFN-α (**a**), IL-1β (**b**), IL-4 (**c**), IL-6 (**d**), IL-12 (**e**), IL-10 (**f**), and TNF (**g**) from PAMs supernatant were measured by Luminex-based multiplex assay. (**h-i**) PAMs were stimulated with different doses of HI-Ro (MOI 100, 50, 25, 5, and 1), Frac-Ro (dilution 1/2, 1/4, 1/8, 1/16, 1/32), LPS (1 μg/mL), or left untreated for 24 h. The percentage of viable cells was determined by flow cytometry. Non-stimulated cells were used as control. Significant differences were determined using a one-way ANOVA (* p-value ≤ 0.05).

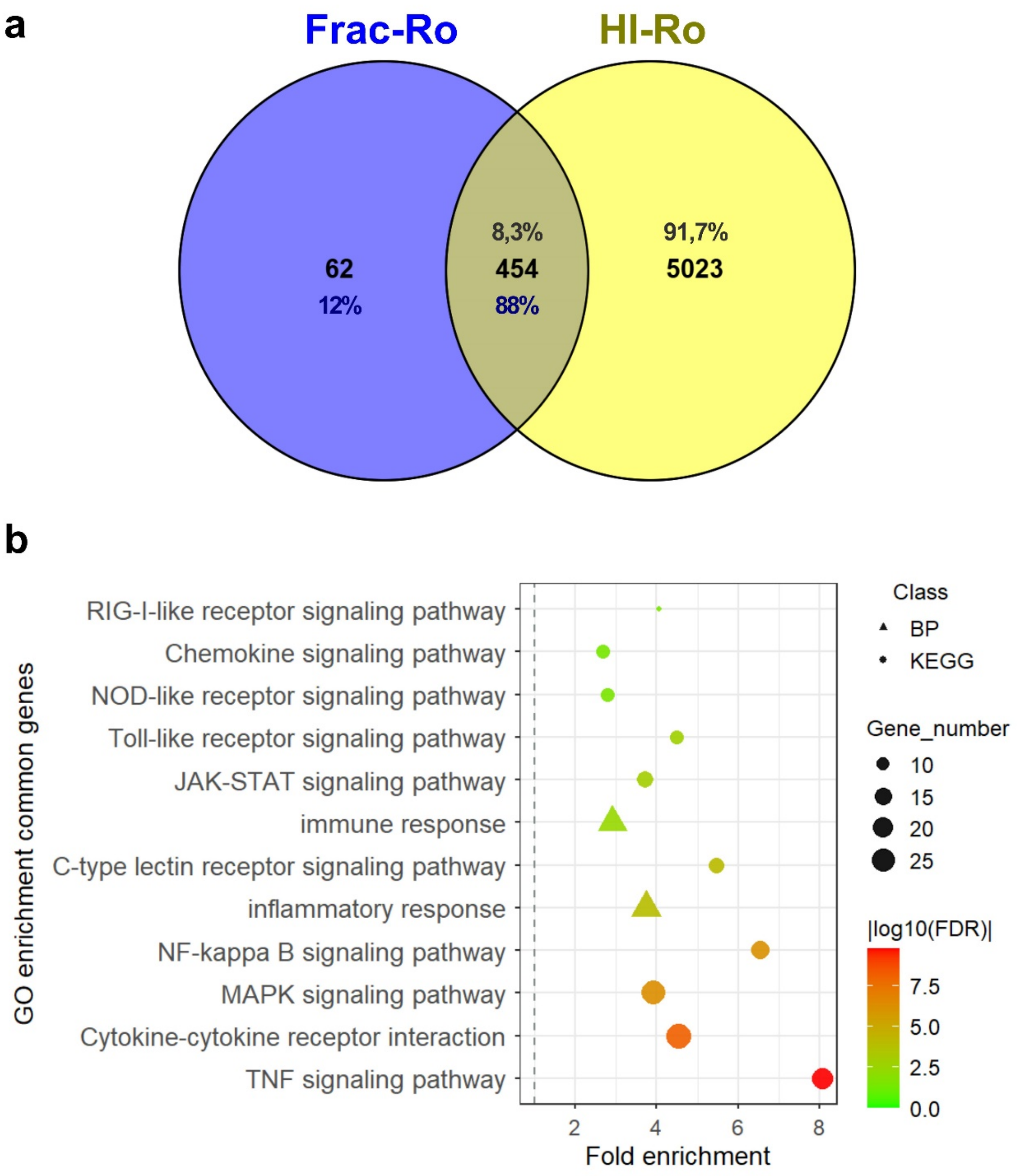

Supplementary Figure 2. (**a**) Venn diagram showing the number and proportions of differentially expressed (DE) genes in macrophages stimulated for 6 hours with Frac-Ro (blue) or HI-Ro (yellow). The upper percentages indicate the proportion of DE genes from HI-Ro-treated cells that overlap with those from Frac-Ro-treated cells. The bottom percentages indicate the proportion of DE genes from Frac-Ro-treated cells that overlap with those from HI-Ro-treated cells. (**b**) Representative Gene Ontology (GO) terms enriched among the 454 overlapping DE genes.

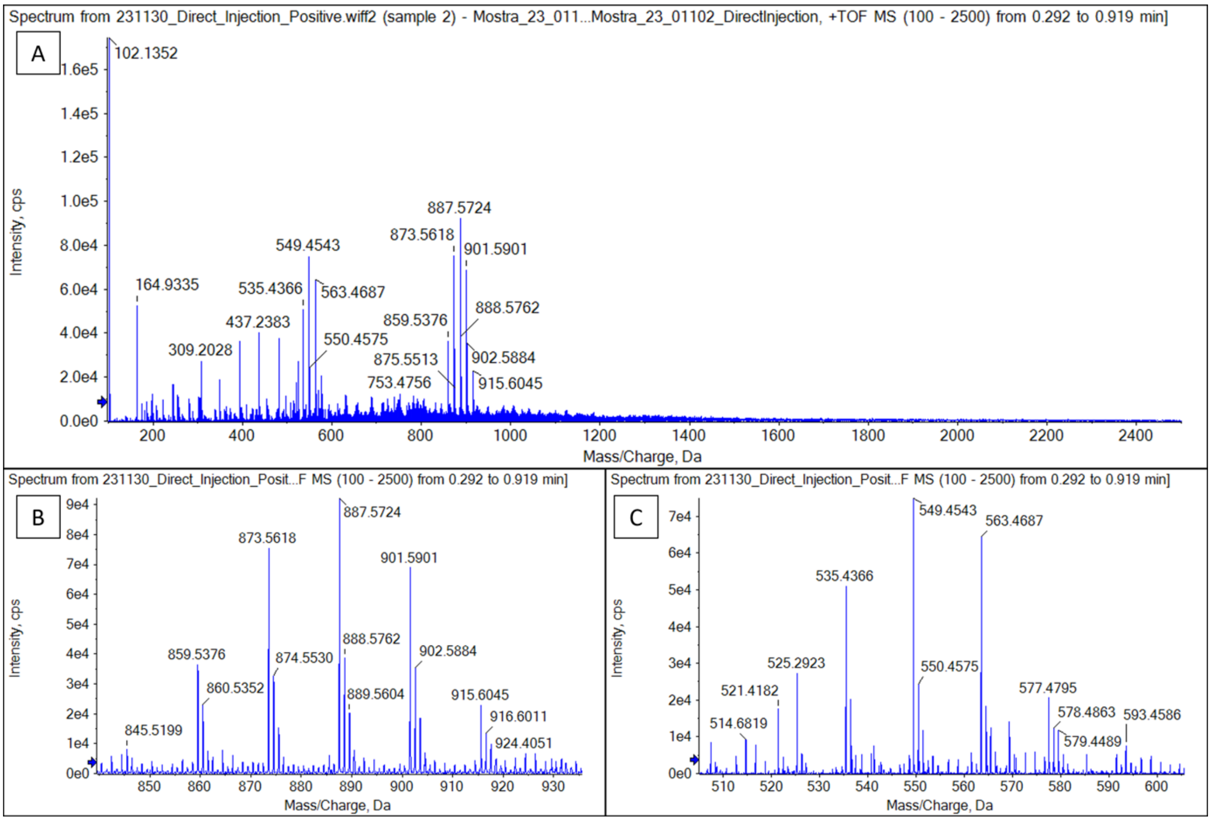

Supplementary Figure 3. High-resolution mass spectrometric characterization of Frac-Ro. (**a**) Direct infusion UHPLC-QTOF MS analysis of the Frac-Ro bacterial culture supernatant fraction acquired in ESI positive mode (m/z 100–2500) revealed two predominant ion clusters in the 500–600 Da and 800–900 Da regions. (**b**) Expanded view of the 800–900 Da region showing a homologous series of ions separated by 14.01 Da, consistent with incremental, CH_2 extensions in aliphatic chains. The major ions (e.g., m/z 915.6042, 901.5911, 887.5731, 873.5629) were assigned as $[M+Na]^+$ adducts with mass errors <10 ppm, supporting the presence of structurally related glycolipids differing in fatty acyl chain length. (**c**) Expanded view of the 500–600 Da region. Although ions were detected in this range, their mass errors exceeded 10 ppm relative to proposed sodium adducts, preventing confident assignment to the same glycolipid family. All spectra were acquired following filtration of the sample through a 0.22 µm membrane and analyzed using SWATH® acquisition for MS and MS/MS data.

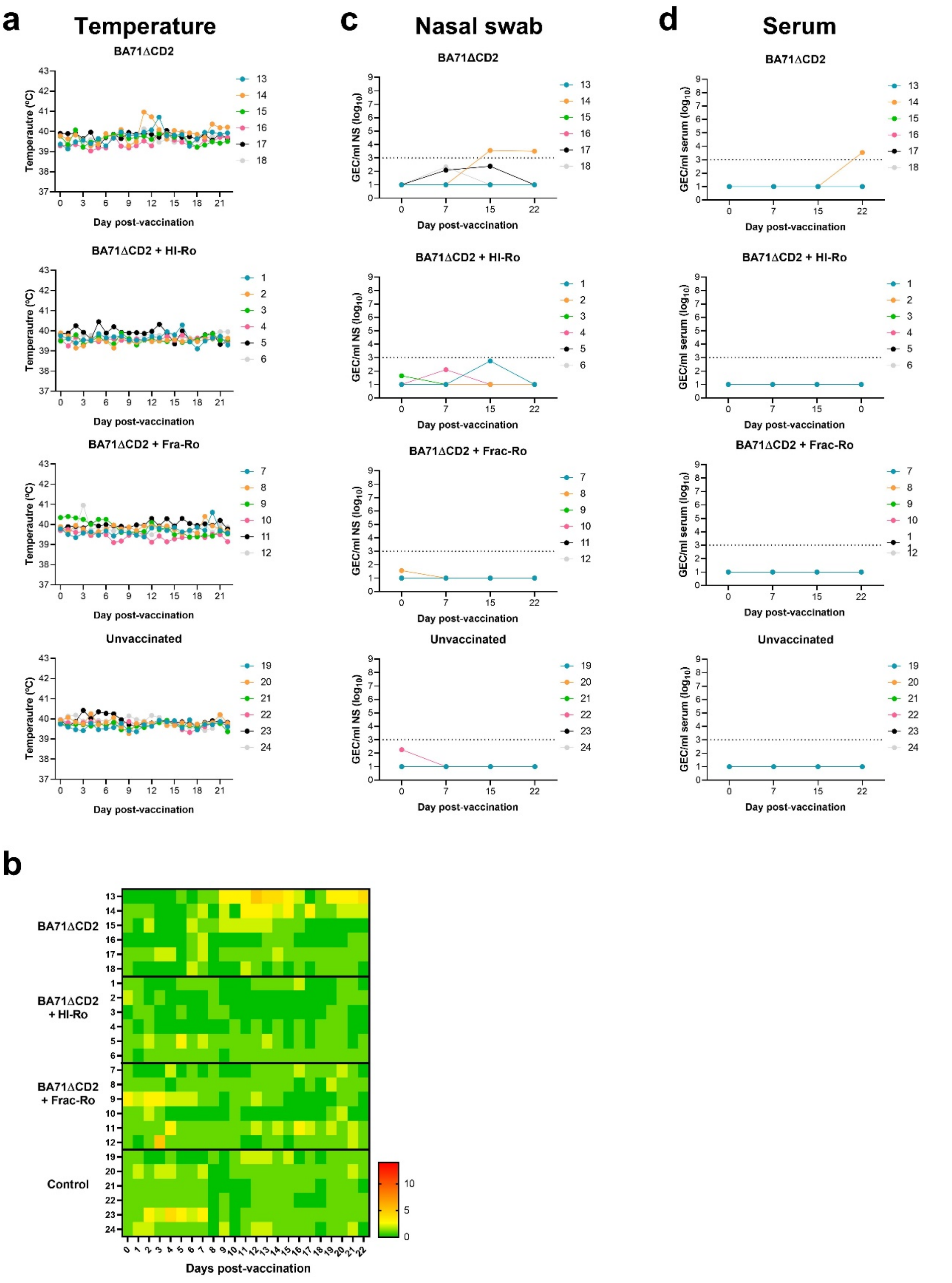

Supplementary Figure 4. Daily rectal temperature charts (**a**) and clinical scores (**b**) of individual pigs from day 0 to day 22 post-vaccination (p.v.). The severity of the clinical signs (score value) is represented by a coloured gradient: green (absence), yellow (mild), and orange/red (severe). (**c-d**) Virus loads measured by qPCR in nasal swabs (**c**) and serum (**d**) at days 0, 7, 15, and 22 p.v. Genomic equivalent copies (GEC) of ASFV were quantified by the detection of the ASFV gene *PK*.

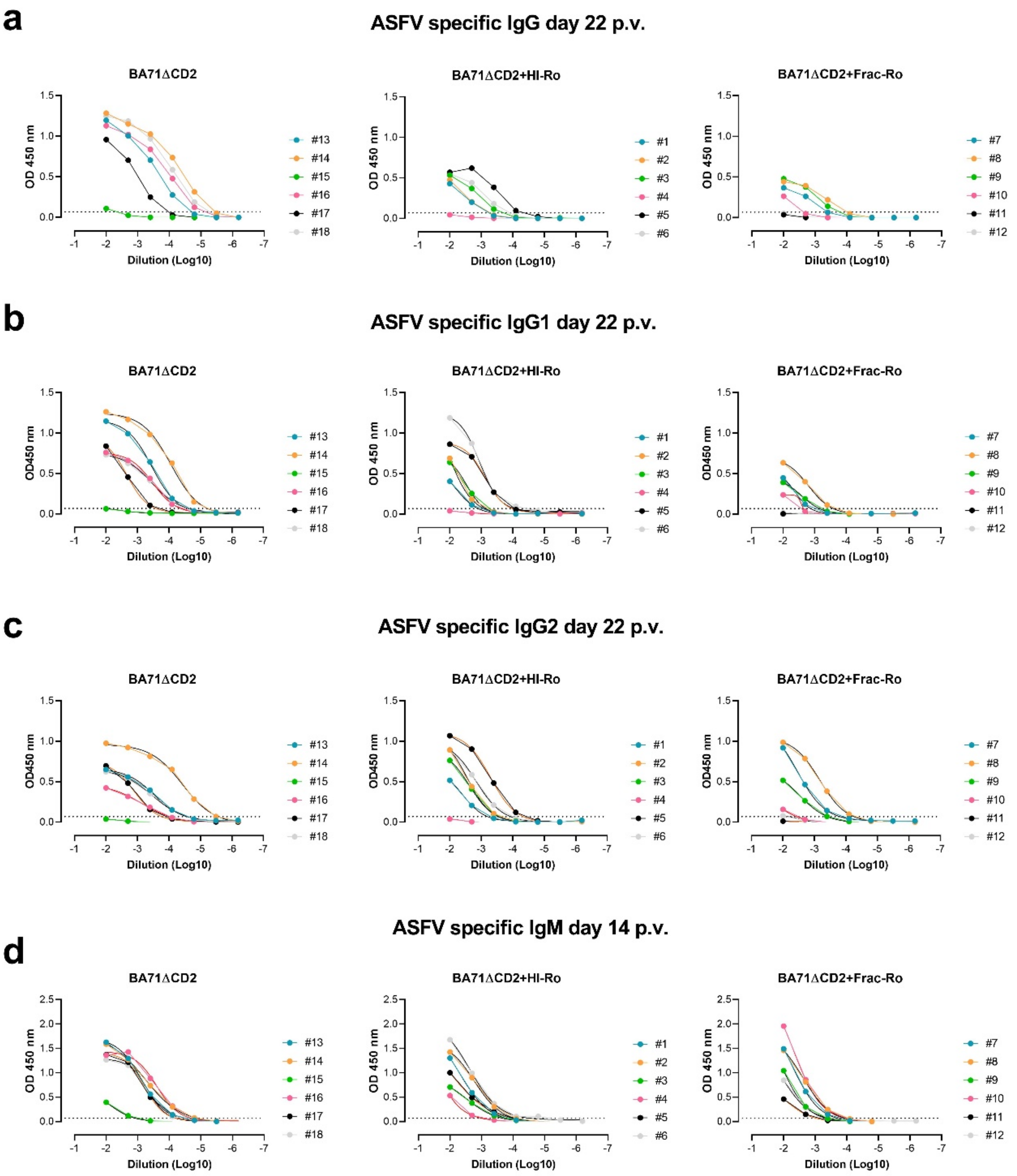

Supplementary Figure 5. ASFV-specific IgG (**a**), IgG1 (**b**), IgG2 (**c**), and IgM (**d**) antibodies measured by ELISA at 22 (**a-c**) or 14 (**d**) days p.v. for each animal.

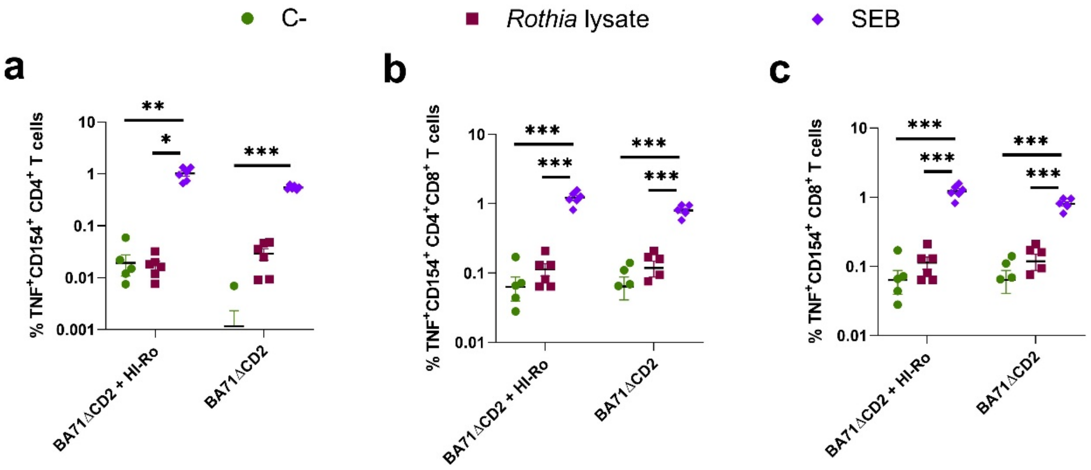

Supplementary Figure 6. PBMCs collected at day 0 post-challenge were stimulated with *Rothia* lysate (10 μg/mL), SEB (2ug/mL), or left unstimulated. After 8 hours of stimulation, the percentage of CD4$^+$, CD8α$^+$, and CD4$^+$CD8α$^+$ T cells co-expressing TNF and CD154 was measured by flow cytometry. Significant differences were determined using a one-way ANOVA (* p-value ≤ 0.05, **≤ 0.01, ***≤ 0.001).

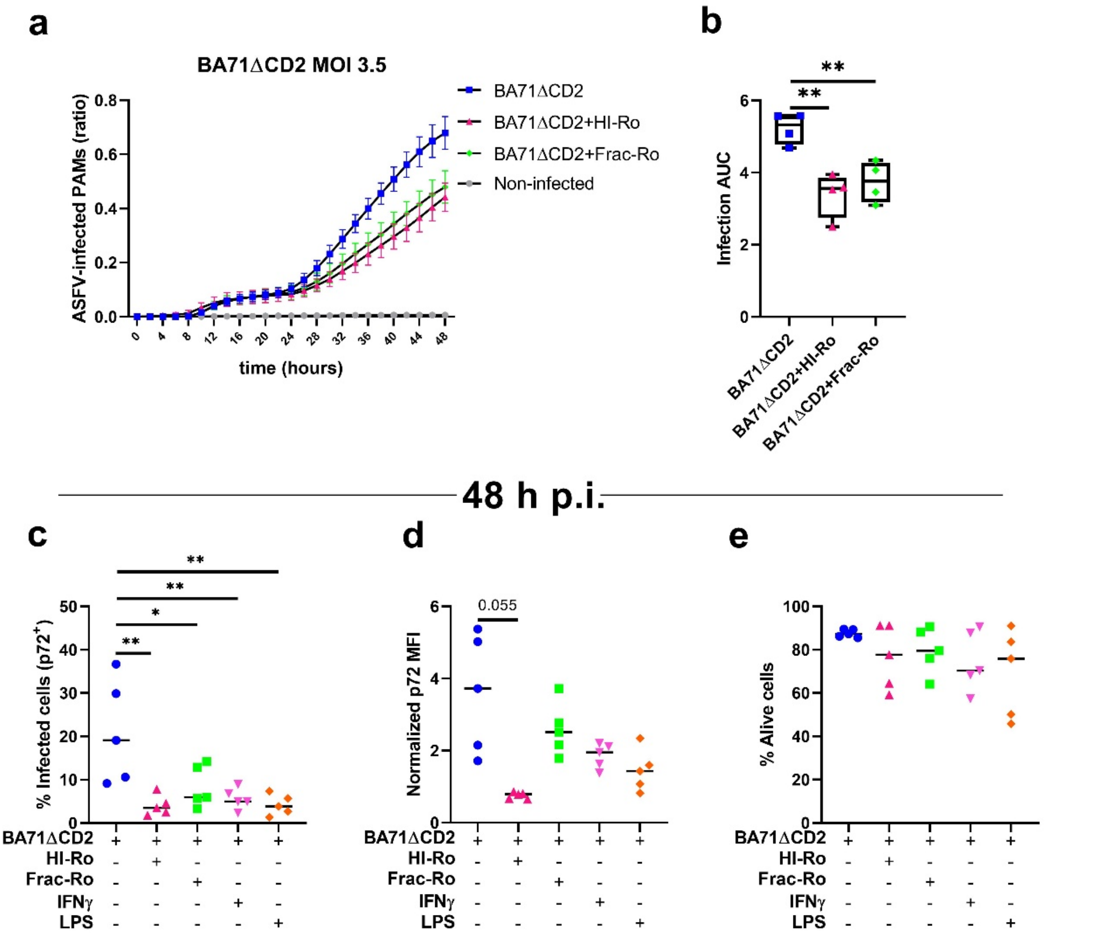

Supplementary Figure 7. (**a-b**) PAMs were infected with the fluorescently-labelled BA71ΔCD2-mWasabi at MOI 3.5. Two hours (h) post-infection, cells were treated with HI-Ro (MOI 50), Frac-Ro or left untreated. (**a**) The percentages of p72-positive infected cells were analyzed every 2 h using time-lapse Incucyte analysis. (**b**) Area under the curve (AUC) for each condition obtained from the kinetics of percentages of infected cells. (**c-e**) BA71ΔCD2-infected PAMs (MOI 3.5) treated with HI-Ro (MOI 50), Frac-Ro, IFN-γ (0.1 μg/mL), LPS (10 μg/mL) or left untreated were analyzed at 72 h post-infection by flow cytometry. The percentages of BA71ΔCD2-infected cells (**c**), the mean fluorescence intensity (MFI) of the p72 ASFV protein (**d**), and the percentage of alive cells (**e**) are shown. Non-infected cells were used as control. Significant differences were determined using a one-way ANOVA (* p-values ≤ 0.05, ** ≤ 0.01, *** ≤ 0.001).

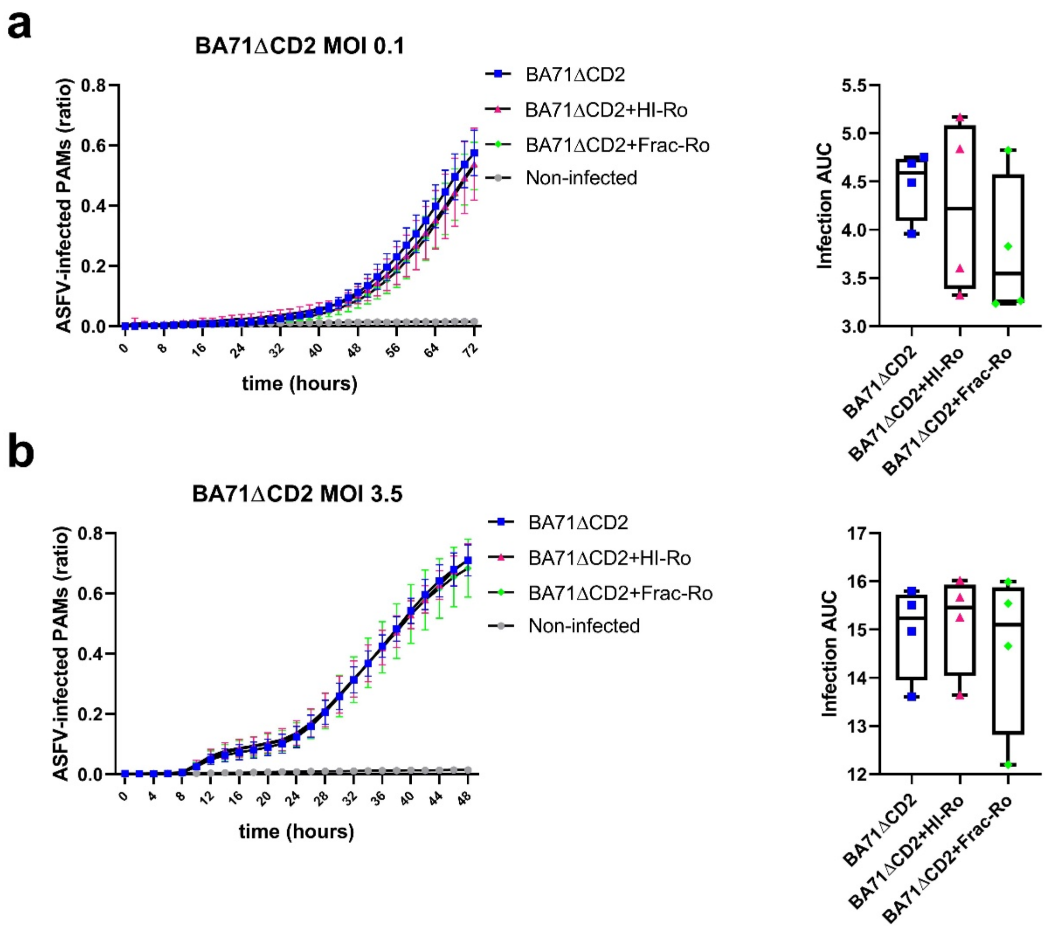

Supplementary Figure 8. Short HI-Ro or Frac-Ro stimulation do not impair BA71ΔCD2 replication. PAMs were stimulated with HI-Ro (MOI 50), Frac-Ro or left untreated for 2 hours (**a-b**). Subsequently, cells were infected with the fluorescent-labelled BA71ΔCD2-mWasabi at MOI 0.1 (**a**) or 3.5 (**b**). The percentages of ASFV-infected cells quantified every 2 hours using time-lapse Incucyte analysis, and the area under the curve (AUC) of the virus kinetics expansion was calculated.

Supplementary Data 1. Statistical analysis results from microfluidic quantitative PCR assay data.

Gene	group1	group2	n1	n2	p	p.signif
BCL2	BA71dCD2	Uninfected	5	5	0,497	ns
BCL2	BA71dCD2_Frac-Ro	Uninfected	5	5	0,0673	ns
BCL2	BA71dCD2_HI-Ro	Uninfected	5	5	0,000835	***
CASP8	BA71dCD2	Uninfected	5	5	0,678	ns
CASP8	BA71dCD2_Frac-Ro	Uninfected	5	5	0,923	ns
CASP8	BA71dCD2_HI-Ro	Uninfected	5	5	0,0373	*
CCL2	BA71dCD2	Uninfected	5	5	0,897	ns
CCL2	BA71dCD2_Frac-Ro	Uninfected	5	5	0,0358	*
CCL2	BA71dCD2_HI-Ro	Uninfected	5	5	0,141	ns
CCL3	BA71dCD2	Uninfected	5	5	0,893	ns
CCL3	BA71dCD2_Frac-Ro	Uninfected	5	5	0,00157	**
CCL3	BA71dCD2_HI-Ro	Uninfected	5	5	0,000159	***
CCL4	BA71dCD2	Uninfected	5	5	0,791	ns
CCL4	BA71dCD2_Frac-Ro	Uninfected	5	5	0,000497	***
CCL4	BA71dCD2_HI-Ro	Uninfected	5	5	0,0000853	****
CCL5 (RANTES)	BA71dCD2	Uninfected	5	5	0,766	ns
CCL5 (RANTES)	BA71dCD2_Frac-Ro	Uninfected	5	5	0,458	ns
CCL5 (RANTES)	BA71dCD2_HI-Ro	Uninfected	5	5	0,0498	*
CCR2	BA71dCD2	Uninfected	5	5	0,532	ns
CCR2	BA71dCD2_Frac-Ro	Uninfected	5	5	0,0198	*
CCR2	BA71dCD2_HI-Ro	Uninfected	5	5	0,132	ns
CCR7	BA71dCD2	Uninfected	5	5	0,688	ns
CCR7	BA71dCD2_Frac-Ro	Uninfected	5	5	0,287	ns
CCR7	BA71dCD2_HI-Ro	Uninfected	5	5	0,00216	**
CD80	BA71dCD2	Uninfected	5	5	0,876	ns
CD80	BA71dCD2_Frac-Ro	Uninfected	5	5	0,862	ns
CD80	BA71dCD2_HI-Ro	Uninfected	5	5	0,0388	*
CXCL2	BA71dCD2	Uninfected	5	5	0,891	ns
CXCL2	BA71dCD2_Frac-Ro	Uninfected	5	5	0,0338	*
CXCL2	BA71dCD2_HI-Ro	Uninfected	5	5	0,12	ns
CXCL9	BA71dCD2	Uninfected	5	5	0,504	ns
CXCL9	BA71dCD2_Frac-Ro	Uninfected	5	5	0,309	ns
CXCL9	BA71dCD2_HI-Ro	Uninfected	5	5	0,0013	**
IFNA1	BA71dCD2	Uninfected	5	5	0,731	ns
IFNA1	BA71dCD2_Frac-Ro	Uninfected	5	5	0,118	ns
IFNA1	BA71dCD2_HI-Ro	Uninfected	5	5	0,00000335	****
IFNB	BA71dCD2	Uninfected	5	5	0,224	ns
IFNB	BA71dCD2_Frac-Ro	Uninfected	5	5	0,59	ns
IFNB	BA71dCD2_HI-Ro	Uninfected	5	5	0,0475	*
IL12A	BA71dCD2	Uninfected	5	5	0,976	ns
IL12A	BA71dCD2_Frac-Ro	Uninfected	5	5	0,0722	ns
IL12A	BA71dCD2_HI-Ro	Uninfected	5	5	0,000714	***
IL12B	BA71dCD2	Uninfected	5	5	0,918	ns
IL12B	BA71dCD2_Frac-Ro	Uninfected	5	5	0,0549	ns
IL12B	BA71dCD2_HI-Ro	Uninfected	5	5	0,00376	**
IL18	BA71dCD2	Uninfected	5	5	0,726	ns
IL18	BA71dCD2_Frac-Ro	Uninfected	5	5	0,211	ns
IL18	BA71dCD2_HI-Ro	Uninfected	5	5	0,0135	*
IL1B	BA71dCD2	Uninfected	5	5	0,955	ns
IL1B	BA71dCD2_Frac-Ro	Uninfected	5	5	0,108	ns
IL1B	BA71dCD2_HI-Ro	Uninfected	5	5	0,0000928	****
IL23	BA71dCD2	Uninfected	5	5	0,95	ns
IL23	BA71dCD2_Frac-Ro	Uninfected	5	5	0,625	ns
IL23	BA71dCD2_HI-Ro	Uninfected	5	5	0,00009	****
IL27	BA71dCD2	Uninfected	5	5	0,951	ns
IL27	BA71dCD2_Frac-Ro	Uninfected	5	5	0,0111	*
IL27	BA71dCD2_HI-Ro	Uninfected	5	5	0,00148	**
IL6	BA71dCD2	Uninfected	5	5	0,545	ns
IL6	BA71dCD2_Frac-Ro	Uninfected	5	5	0,027	*
IL6	BA71dCD2_HI-Ro	Uninfected	5	5	0,000269	***
IL8	BA71dCD2	Uninfected	5	5	0,98	ns
IL8	BA71dCD2_Frac-Ro	Uninfected	5	5	0,0814	ns
IL8	BA71dCD2_HI-Ro	Uninfected	5	5	0,0000415	****
IRF7	BA71dCD2	Uninfected	5	5	0,823	ns
IRF7	BA71dCD2_Frac-Ro	Uninfected	5	5	0,355	ns
IRF7	BA71dCD2_HI-Ro	Uninfected	5	5	0,0158	*
ISG 15	BA71dCD2	Uninfected	5	5	0,69	ns
ISG 15	BA71dCD2_Frac-Ro	Uninfected	5	5	0,439	ns
ISG 15	BA71dCD2_HI-Ro	Uninfected	5	5	0,0024	**
MX1	BA71dCD2	Uninfected	5	5	0,324	ns
MX1	BA71dCD2_Frac-Ro	Uninfected	5	5	0,295	ns
MX1	BA71dCD2_HI-Ro	Uninfected	5	5	0,013	*
NFKB1	BA71dCD2	Uninfected	5	5	0,738	ns
NFKB1	BA71dCD2_Frac-Ro	Uninfected	5	5	0,566	ns
NFKB1	BA71dCD2_HI-Ro	Uninfected	5	5	0,0326	*
NFKB2	BA71dCD2	Uninfected	5	5	0,549	ns
NFKB2	BA71dCD2_Frac-Ro	Uninfected	5	5	0,65	ns
NFKB2	BA71dCD2_HI-Ro	Uninfected	5	5	0,0319	*
NLRP3	BA71dCD2	Uninfected	5	5	0,2	ns
NLRP3	BA71dCD2_Frac-Ro	Uninfected	5	5	0,203	ns
NLRP3	BA71dCD2_HI-Ro	Uninfected	5	5	0,0973	ns
SLA-DRB	BA71dCD2	Uninfected	5	5	0,994	ns
SLA-DRB	BA71dCD2_Frac-Ro	Uninfected	5	5	0,744	ns
SLA-DRB	BA71dCD2_HI-Ro	Uninfected	5	5	0,602	ns
SLA-DRB1	BA71dCD2	Uninfected	5	5	0,973	ns
SLA-DRB1	BA71dCD2_Frac-Ro	Uninfected	5	5	0,864	ns
SLA-DRB1	BA71dCD2_HI-Ro	Uninfected	5	5	0,472	ns
TNFA	BA71dCD2	Uninfected	5	5	0,911	ns
TNFA	BA71dCD2_Frac-Ro	Uninfected	5	5	0,00949	**
TNFA	BA71dCD2_HI-Ro	Uninfected	5	5	0,000273	***
TLR1	BA71dCD2	Uninfected	5	5	0,292	ns
TLR1	BA71dCD2_Frac-Ro	Uninfected	5	5	0,553	ns
TLR1	BA71dCD2_HI-Ro	Uninfected	5	5	0,481	ns

Supplementary data 2. Sequence of primers used for microfluidic qPCR assays.

Gene	Ensamble code	Forward primer	Reverse primer	R2	% Efficiency
IL-1B	ENSSSCG00000039214	TCTCTCACCCCTTCTCCTCA	GACCCTAGTGTGCCATGGTT	88	0,997
IL-6	ENSSSCG00000020970	CCTCTCCGGACAAAACTGAA	TCTGCCAGTACCTCCTTGCT	72	0,978
IL-12A	ENSSSCG00000011730	CCACCTGGACCATCTCAGTT	CAGCAGATTTTGGGAGTGGT	94	0,965
IL-12B	ENSSSCG00000017044	GACCAGAAAGAGCCCAAAAAC	AGGTGAAACGTCCGGAGTAA	78	0,991
IL-18	ENSSSCG00000015037	CTGCTGAACCGGAAGACAAT	TCCGATTCCAGGTCTTCATC	80	0,997
IL-23	ENSSSCG00000033520	CAACAGTCAGTCCTGCTTGC	GCTCCCCTGTGAAAATGTCT	87	0,995
IL-27	ENSSSCG00000039300	TCTGAGGCTGAATCACACCTGC	GAGTCGTTCTGGGTCAGAGAGG	87	0,995
TNF	ENSSSCG00000060860	ACTGCACTTCGAGGTTATCGG	TGAGACGATGATCTGAGTCCT	86	0,988
NLRP3	ENSSSCG00000013940	GACTTTCCAGGAGTTCTTTGCTG	CCTGGTTTACAAGGCCAAAG	86	0,99
CCL2	ENSSSCG00000017723	ACCAGCAGCAAGTGTCCTAAA	GTCCAGGTGGCTTATGGAGT	87	0,996
CCL3	ENSSSCG00115008383	CTCTGCAGCCAGGTCTTCTC	CTACGAATTTGCGAGGAAGC	87	0,993
CCL4	ENSSSCG00000032343	TCCTCGCAACTTCGTGACTG	CCTTTTTGGTCTGGAATACCACGG	85	0,998
CCL5 (RANTES)	ENSSSCG00000017705	CTCCATGGCAGCAGTCGT	AAGGCTTCCTCCATCCTAGC	89	0,996
IL-8	ENSSSCG00000008953	TTGCCAGAGAAATCACAGGA	TGCATGGGACACTGGAAATA	101	0,988
CXCL2	ENSSSCG00000008959	GGATAGCACGCTGTACCATC	ACTGTCTCAATAAATAACAACCGAC	88	0,997
CXCL9	ENSSSCG00000032777	TGATTGGAGTTCAAGGAACCCT	TCTCACAAGAAGGGCTTGGG	81	0,986
CCR2	ENSSSCG00000024311	AAGAAGCAACAGACCGGGCA	CTTCGATTTGTCCCACGCTG	60	0,985
NFKB1	ENSSSCG00000030957	TCCACAAGGCAGCAAATAGA	AAGCTGAGTTTGCGAAAGGA	86	0,995
NFKB2	ENSSSCG00000010580	GAACAGCCTAAGCAGCGAGG	GCCCTTCTCACTAGAGGCAC	83	0,989
IFNA1	ENSSSCG00000052950	TTCCAGCTCTTCAGCACAGA	AGCTGCTGATCCAGTCCAGT	88	0,978
IRF7	ENSSSCG00000012853	GGCACCTTCTTCCAAGAGTTGAT	GAAGCCCAGGTAGATGGTGTAG	87	0,994
MX1	ENSSSCG00000012077	AGACAGGACCATCGGGATCTT	TTCTCGCCACGTCCACTATC	88	0,996
ISG15	ENSSSCG00000040575	GGACCTGAAGCAGCAGATCG	CACCCCATCCTGAAGCACAT	90	0,995
CD80	ENSSSCG00000011899	CGCACCTTCACTGATGTCAC	CACAGGTGTAGGTGCCATTG	80	0,981
CCR7	ENSSSCG00000017466	GGTCCAACCAACTCTCGGAAT	TTGCTGGCTCGGATGACA	86	0,989
SLA-DRB1	ENSSSCG00000001455	TGACGGTGTATCCTGCAAAG	GTAGAACCCGGTCACAGAGC	77	0,993
TLR1	ENSSSCG00000026583	CCTTCAAGACCTTAACACACAGAG	CAGATTTACTGCGGTGCTGA	78	0,998
IFNB	ENSSSCG00000005163	CACTGGCTGGAATGAAACCG	AATGGTCATGTCTCCCCTGG	61	0,996
CASP8	ENSSSCG00000028157	CCGAAACTTGGACCATAATGA	GATGATGCCCTTGTCTCCAT	89	0,995
BCL2	ENSSSCG00000062696	CCCTGTGGATGACTGAGTACC	AACCACACATGCACCTACCC	100	0,983
ACTB	ENSSSCG00000007585	CTACGTCGCCCTGGACTTC	GCAGCTCGTAGCTCTTCTCC	94	0,999
YWHAZ	ENSSSCG00000006062	ATTGGGTCTGGCCCTTAACT	GCGTGCTGTCTTTGTATGACTC	89	0,966
RPL4	ENSSSCG00000004945	AGGAGGCTGTTCTGCTTCTG	TCCAGGGATGTTTCTGAAGG	90	0,997
GAPDH	ENSSSCG00000000694	GTGCATTGCCAGCCGCGT	ACTGGAACATGTAGACCATGTAGT	86	0,997
